# Connecting the Dots: a cluster-randomized clinical trial integrating standardized autism spectrum disorders screening, high-quality treatment, and long-term outcomes

**DOI:** 10.1186/s13063-021-05286-6

**Published:** 2021-05-02

**Authors:** Leslie A. McClure, Nora L. Lee, Katherine Sand, Giacomo Vivanti, Deborah Fein, Aubyn Stahmer, Diana L. Robins

**Affiliations:** 1 Drexel University, 3215 Market Street, Philadelphia, PA 19104 USA; 2 AJ Drexel Autism Institute, Drexel University, 3020 Market Street, Philadelphia, PA 19104 USA; 3 University of Connecticut, 406 Babbidge Rd, Storrs, CT 06268 USA; 4 University of California, Davis, 2825 50th St, Sacramento, CA 95819 USA

**Keywords:** Autism spectrum disorder, Toddler screening, M-CHAT-R/F

## Abstract

**Background:**

Autism spectrum disorder (ASD) affects one in 54 children in the United States of America, and supporting people with ASD across the lifespan presents challenges that impact individuals, families, and communities and can be quite costly. The American Academy of Pediatrics has issued recommendations for routine ASD screening at 18 and 24 months, but some research suggests that few pediatricians perform high-fidelity, standardized screening universally. Furthermore, the United States Preventive Services Task Force (USPSTF) found insufficient evidence to recommend for or against universal ASD screening. The objective of this study is to test the hypothesis that children with ASD who have high fidelity; standardized screening will achieve superior outcomes at 5 years of age compared to children receiving usual care ASD detection strategies.

**Methods:**

This is a cluster-randomized, controlled clinical trial in 3 sites in the USA. Pediatric practices will be randomized to implement universal, standardized, high-fidelity toddler screening or usual care, with randomization stratified by the practice size. The study will enroll 3450 children, approximately half in each group. From this sample, we anticipate 100 children to be diagnosed with ASD. Children in both groups receiving an ASD diagnosis will be administered the Early Start Denver Model, an evidence-based early intervention addressing social, communication, and cognitive functioning. Treatment will last for 1 year, with up to 20 h per week of therapy for children with ASD.

**Results:**

Primary outcomes measured at baseline, following treatment, and at 4 and 5 years of age include ASD symptom severity (Brief Observation of Social Communication Change (BOSCC)) and cognitive functioning (Mullen Scales of Early Learning (MSEL) and Differential Abilities Scale-II (DAS-II)). Secondary outcomes in children include measures of adaptive functioning, ASD symptoms, and kindergarten readiness; secondary analyses will also examine stress and empowerment among parents. Several novel exploratory measures will be included as well. The study will utilize a modified intention-to-treat analysis.

**Conclusions:**

This trial will evaluate the impact of universal, standardized, high-fidelity screening for ASD among children at 18 months of age, with a goal of providing evidence to support this strategy to detect ASD in toddlers in order to start treatment as young as possible and maximize outcomes.

**Ethics and dissemination:**

This study was approved by the Institutional Review Board at Drexel University (IRB protocol: 1607004653). All findings will be provided by the principal investigator via email; data will be available through the NIMH Data Archive (https://nda.nih.gov/).

**Trial registration:**

ClinicalTrials.gov NCT03333629. Registered on November 7, 2017

**Supplementary Information:**

The online version contains supplementary material available at 10.1186/s13063-021-05286-6.

## Background

Autism spectrum disorder (ASD) affects one in 54 children in the USA [1]. Supporting people with ASD across the lifespan includes addressing challenges with education, employment, and independent living which can be costly at the individual, family, and societal level. Overall, ASD is associated with $3.6 million in pro-capita lifetime social cost [2], exceeding the costs of both stroke and hypertension.

ASD-specific intervention can change life course trajectories of children with ASD, improving quality of life and self-determination in children and adults, ameliorating family well-being, and reducing societal costs [3–5]. Early interventions with substantial empirical evidence for their effectiveness include Early Intensive Behavioral Intervention which is based on the adult-directed approach known as discrete trial training [6], as well as Naturalistic Developmental Behavioral Interventions [7], which incorporate child-led teaching episodes within naturally occurring contexts and contingencies. Treatment effects for these interventions, as documented in several randomized controlled trials, include gains in cognitive, adaptive, and social communication functioning, both immediately after treatment [8–10] and several years after treatment cessation [11, 12]. Furthermore, recent research indicates that children who begin treatment earlier in life achieve better outcomes compared to those who delay treatment onset [6, 13–16]. However, detecting ASD as young as possible is challenging.

**Fig. 1:**
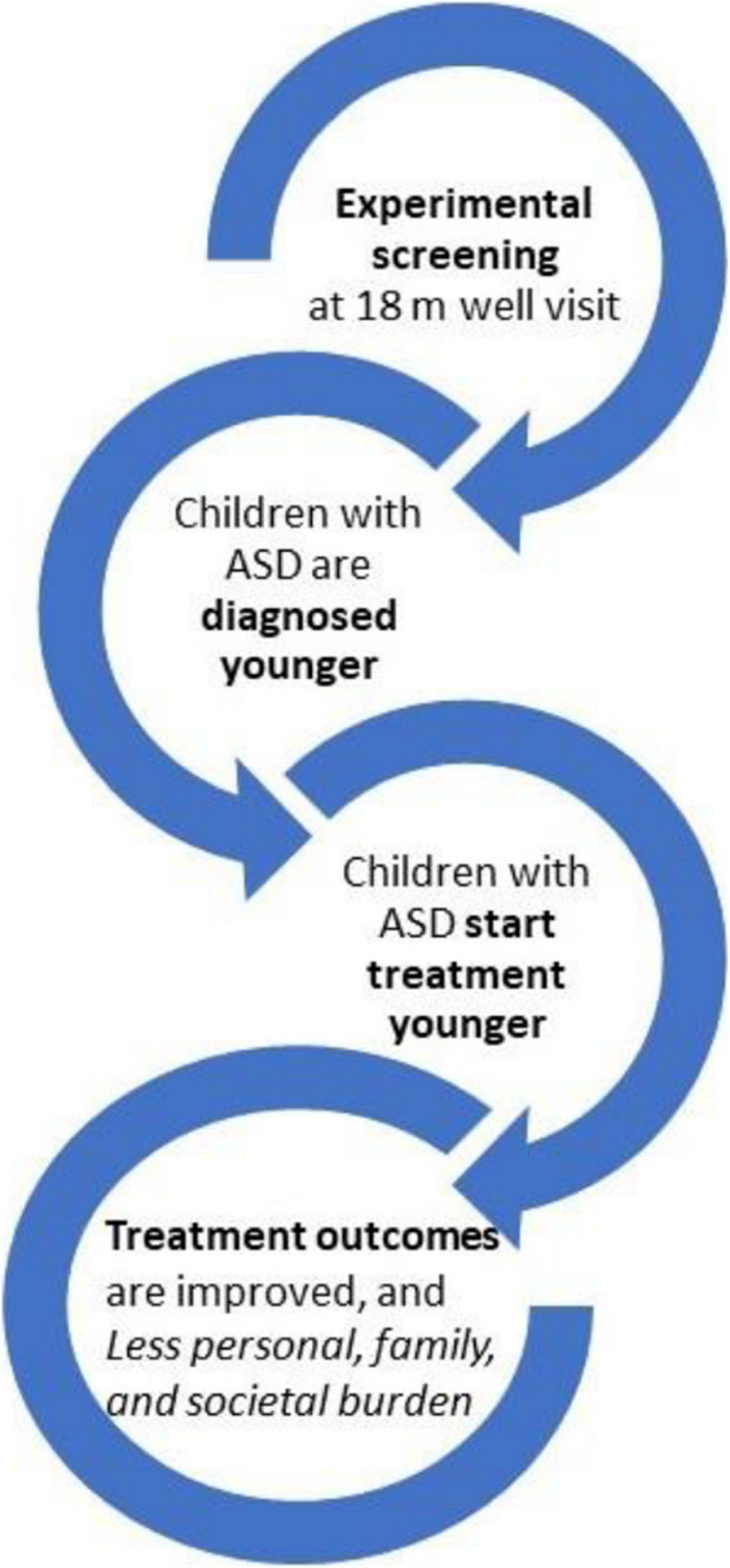
Conceptual model supporting the study hypothesis

One strategy for identifying a high likelihood of ASD in young children is routine screening in the context of pediatric health care. The most widely used ASD screening tool during well-child visits is the Modified Checklist for Autism in Toddlers (M-CHAT) [17], and its revision, the M-CHAT Revised, with Follow-Up (M-CHAT-R/F) [18], consisting of 20 questions completed by parents, and targeted follow-up for at-risk responses when indicated. In an unselected sample of more than 16,000 children screened in well-child visits (mean age=20.9 months, SD=3.3), the M-CHAT-R/F demonstrated adequate psychometrics when diagnostic evaluation is done shortly after screening: sensitivity=.83, specificity=.99, positive predictive value (PPV)=.45, and negative predictive value (NPV)=.99 [19]. Although some prospective studies based on clinical record review have reported lower sensitivity [20, 21], there is substantial evidence that implementation of the M-CHAT-R/F, coupled with immediate referrals for treatment, can lower the average age of diagnosis by 2 years [19] and reduce disparities in the age of diagnosis [22]. This finding, along with evidence that starting treatment at a younger age is associated with improved outcomes, suggests that toddler screening has the potential to improve the lives of individuals with ASD (see Fig. 1).

Direct evidence for this impact of screening on outcomes is still incomplete. Based on an analysis of the existing literature, the US Preventive Services Task Force (USPSTF) indicated that the evidence for improved outcomes with ASD screening in the general pediatric population is not sufficient to recommend universal screening as a standard of care [23]. In particular, they highlighted the lack of randomized controlled trials (RCTs) testing the outcomes of children detected through screening and referred to treatment compared to children detected in other ways. Thus, the current Connecting the Dots study is designed to address this gap in knowledge through an RCT examining the effects of toddler universal, standardized, high-fidelity screening for ASD versus usual care (often non-standardized or low-fidelity screening and/or physician surveillance) on short- and long-term outcomes.

Since the American Academy of Pediatrics (AAP) issued its initial recommendation for routine ASD screening at 18 and 24 months [24], which was reiterated in 2020 [25], ASD screening has become more prevalent. A 2004 survey of 471 pediatricians found that only 8% screened for ASD [26], compared to more than 50% in a 2011 survey of 406 pediatricians [27]. In a more recent survey of 223 pediatricians, 27% reported routinely screening for ASD following AAP guidelines, while 53% reported screening but did not follow the guidelines [28]. Recent research provides further evidence that, while more pediatricians are using tools like the M-CHAT, the M-CHAT is often not used as intended, including selective administration to specific children (e.g., children presenting noticeable red flags), selective referrals of children who screen positive, and incomplete administration [21, 29]. Incomplete administration of the M-CHAT likely leads to lower sensitivity and specificity, while selective screening and referrals may contribute to disparities in the identification of at-risk children who are racial and ethnic minorities [22]. In the current study, pediatric providers assigned to implement universal, standardized, high-fidelity screening for ASD are trained, and adherence to the M-CHAT protocol is monitored to ensure fidelity of M-CHAT implementation. The use of fidelity procedures, while very rare in ASD programs, is now widely recognized as a critical factor for bridging the “science to service gap” in the health care literature [30].

We will test the hypothesis that children with ASD in the experimental screening group (universal, standardized, high-fidelity screening) will achieve superior outcomes at 5 years of age compared to children receiving usual care. The latter is often a combination of developmental surveillance and screening that may not be universal, standardized, or high-fidelity. Better outcomes for the intervention (screening) group are expected because children with ASD are detected younger, and high-quality evidence-based treatment is begun earlier, including 1 year of ESDM for all children diagnosed with ASD. We will test our hypothesis through the following aims: (1) compare short- and long-term outcomes between the intervention and usual care groups, including cognitive functioning, ASD symptom severity, adaptive functioning, and kindergarten readiness; (2) evaluate the impact of the intervention on the pediatric provider and practice characteristics (e.g., number and ages of children referred for ASD evaluation, provider attitudes and beliefs about screening), as well as caregiver characteristics (e.g., stress and empowerment indicators); (3) evaluate the moderators of the intervention effect on short- and long-term outcomes, including baseline symptom severity, cognitive functioning, and socioeconomic status.

## Methods/design

### Study setting

Three clinical sites contribute to the Connecting the Dots study: Drexel University (Philadelphia, PA), University of Connecticut (Storrs, CT), and the MIND Institute at UC Davis (Sacramento, CA). Pediatric providers who offer primary care to children under 3 years old and located within a 1-h drive will be recruited to participate in the study. The participating providers recruit and enroll children within their practice; children identified at risk for ASD are invited to complete diagnostic testing in the university clinics. Treatment for participating children diagnosed with ASD with Early Start Denver Model (ESDM) occurs in the child’s home, childcare setting, or university clinic.

### Study design, allocation procedures, and blinding

The Connecting the Dots study is a multi-site, cluster-randomized, controlled trial in which participants are assigned to receive either universal, standardized, high-fidelity toddler screening or usual care. Pediatric practices, clustered within study site (Drexel University, UC Davis, UConn), were randomized to either (1) administer universal, standardized, high-fidelity screening for ASD (at 18-month well-child visits) using the Modified Checklist for Autism in Toddlers, Revised, with Follow-Up (M-CHAT-R/F) coupled with provider surveillance, or (2) to detect children at risk for ASD via usual practices. Randomization was stratified by practice size and performed by the Data Coordinating Center using computer-generated random numbers. While only the experimental practices will universally screen at 18-month visits, a child can be referred for ASD risk based on the provider’s clinical judgment anytime between 12 and 48 months old. All eligible children in both practice groups will then be screened for ASD at 48 months of age. A total of 100 children newly diagnosed with ASD will be enrolled in the treatment phase of the study, identified from 3450 children screened. It is anticipated that those in the experimental group will enroll at approximately 18 months of age, while those in the usual care group will enroll at approximately 48 months of age, with some deviation in the age of diagnosis in each group due to missed cases (experimental group) or provider concerns (usual care) early on.

Children who screen positive, or for whom the pediatric provider has ASD concerns, are referred for diagnostic testing. Those who are diagnosed with ASD are then offered 12 months of intensive therapy using the Early Start Denver Model (ESDM) [31], administered by the study staff or outside trained therapists supervised by the study personnel certified as ESDM trainers. Clinical personnel who conduct the diagnostic evaluations and treatment are blinded to the group and, when possible, to the study hypotheses. We do not foresee any reason to unblind these study personnel.

The primary outcomes are 12-month measures of symptom severity, as measured by the Brief Observation of Social Communication Change (BOSCC) [32], and cognitive function, as measured by the Mullen Scales of Early Learning (MSEL) [33]. The secondary outcomes include autism symptomatology, as measured by the Autism Diagnostic Observation Schedule-2 (ADOS-2) [34] and the Pervasive Developmental Disorder Behavior Inventory (PDDBI) [35]; adaptive functioning, as measured by the Vineland Adaptive Behavior Scales (VABS-3) [36]; and kindergarten readiness at 5 years of age, as measured by the Developmental Indicators for the Assessment of Learning, Fourth Edition (DIAL-4) [37]. In addition, exploratory outcomes include measures of changes directly relevant to the social reciprocity processes targeted by treatment, such as eye-tracking experimental paradigms [38], and parent-child social engagement measures [39, 40], and detailed in the “Study measures” section. Finally, parent report of ASD symptoms was collected to characterize the sample, using the Toddler ASD Symptom Inventory (TASI) [41] and the Autism Diagnostic Interview, Revised [42].

The study has been approved by the IRB at Drexel University and is registered with ClinicalTrials.gov (NCT03333629).

### Recruitment and eligibility

Enrollment in the Connecting the Dots study is two-tiered: pediatric practices are recruited to participate in the study, and providers recruit and enroll into the study eligible children who are seen in their practice. Pediatric providers are eligible for inclusion if they are located within a 1-h driving radius of the local university clinic and if they regularly see toddlers for well-child visits. Those who are already using universal, standardized, high-fidelity screening in their practices are not eligible to participate in the study. Eligibility is determined based on an interview with at least one provider from the practice. Providers are asked seven questions about the screening and detection of ASD in toddlers. If a practice is already using universal, standardized, high-fidelity screening practices, including referring all screen-positive cases for ASD evaluation, they are ineligible for participation, as they are already exhibiting best practices in screening for ASD.

Children are eligible for study enrollment if they meet all of the following criteria: (a) regularly seen in enrolled pediatric practice since 21 months old or younger, (b) legal guardian is fluent in English or Spanish, and (c) for experimental practices, attend a well-child visit between ages 16.00–21.99 months during the enrollment phase of the study, and for usual care practices, have a date of birth consistent with an 18-month visit during the enrollment period. Children are not eligible to enroll in the study if they meet any of the following criteria: (a) are completely blind or deaf, (b) suffer from a severe motor impairment that would preclude standardized testing, or (c) are not within the age range for the enrollment cohort. The total sample of toddlers enrolled at pediatric check-ups may be adjusted to achieve the ultimate target of 100 children diagnosed with ASD. Further, screening rates will be assessed at the practices (see the “Quality control” section).

### Screening and enrollment procedures

#### Experimental group

For eligible children in the experimental group, the M-CHAT-R/F is administered during the 18-month well-child visit, which also represents the period during which children in the experimental group can enroll. Parents/legal guardians are provided a Chromebook to access a secure website that links to our study database, although practices also have the option of providing a link for parents to complete the questionnaire prior to the visit. After creating a password-protected account, consenting electronically, and providing demographic information about themselves and their child, caregivers are then asked to complete the M-CHAT-R/F. This two-stage screening tool has 20 yes/no items that parents complete and has additional follow-up questions to clarify the risk status if the child scores moderate risk (initial score 3–7).

**Fig. 2:**
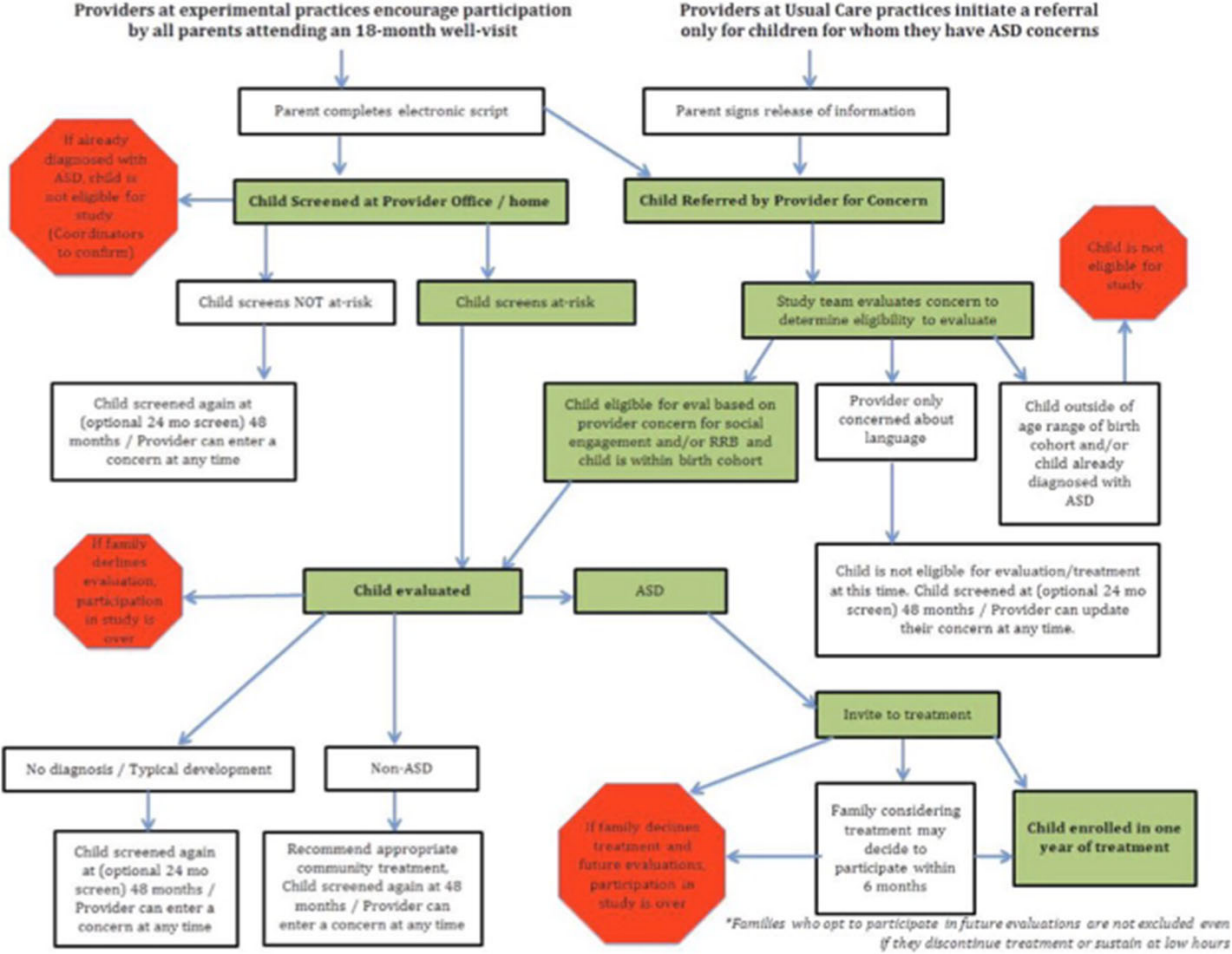
Flowchart of study activities

Caregivers have the option of completing enrollment and screening either in English or Spanish and additionally have the option to listen to recordings of each of the questions and the possible answers, in addition to reading the text. After a provider indicates whether they have ASD concerns about a screened child, the provider can then review the M-CHAT-R/F results. Providers can add new ASD concerns into the system at any time during the child’s participation in the study. M-CHAT-R/F scores and provider concerns are available to the site coordinators for further action, if needed (see Fig. 2 for the flowchart of study activities).

Caregivers for children who screen positive or have provider concerns for ASD are contacted by the site coordinator and offered the opportunity for their child to attend a diagnostic evaluation. Those who agree to the testing attend a 3-h session in the clinic, during which measures of cognition, language, adaptive functioning, and ASD symptomatology are completed by the children and parents. Diagnosis is made by clinical best estimate, considering all available information. Children who are diagnosed with ASD are then invited to participate in 12 months of intensive therapy with the ESDM.

All children who were screened at 18 months will be screened again at 48 months using both the M-CHAT-R/F and the Social Communication Questionnaire (SCQ) [43] to ascertain additional cases that may have been missed at earlier visits, and procedures for diagnosis and treatment will be as described above. Those who decline treatment at 18 months will not be eligible to receive treatment later in the study.

#### Usual care group

Children in the usual care group are eligible for the study if they were seen in the participating practice for a well-child visit by age 21 months or younger and fall into the practice-specific date of birth range established during the enrollment phase. Prior to 48 months, pediatric providers use their clinical judgment to identify ASD concerns in children. Providers fax ASD concerns to the study team. The coordinator then reaches out to the families to invite them for diagnostic testing (should they meet the eligibility criteria). If the child is diagnosed with ASD, they are invited to participate in 12 months of intensive therapy with the ESDM.

All usual care children who are in the appropriate birth cohort and who have been seen at the enrolled practice since age 21 months (or earlier) are eligible to be screened at 48 months for ASD with both the M-CHAT-R/F and the SCQ via our online system, as described above for the experimental group. Thus, recruitment for usual care children is through 48 months of age. Those who screen positive for ASD, or for whom providers express concerns at this time, will be evaluated in the same fashion as described for the experimental group above. Children diagnosed with ASD will be invited to participate in 12 months of intensive therapy with the ESDM.

### Treatment and post-testing

All children who are diagnosed with ASD and who enter treatment will receive up to 20 h per week of ESDM therapy for 12 months. The ESDM is an early intervention approach for children with ASD ages 12 to 48 months that includes a manualized set of treatment procedures and a comprehensive curriculum covering multiple developmental areas [44]. Treatment strategies in the ESDM are based on the Naturalistic Developmental Behavioral Interventions approach. This includes the use of operant conditioning principles to promote the acquisition of social, cognitive, and adaptive skills in the context of naturalistic and socially engaging routines that incorporate child choices and everyday life materials. Intervention targets are informed by research on the developmental sequences and prerequisites for the acquisition of specific skills (e.g., joint attention, imitation, and functional play as key precursors to language). Implementation of the ESDM includes the creation of measurable learning objectives developed from a comprehensive assessment of the child’s behavioral repertoire (the ESDM Curriculum Checklist) [45], with child progress systematically recorded against operationally defined mastery criteria. The ESDM Curriculum Checklist is re-administered every 12 weeks, so that goals can be updated quarterly. In the current project, ESDM is delivered in a 1:1 fashion in the child’s home by clinicians that are trained by a certified ESDM trainer. Additionally, biweekly parent coaching is provided.

All children will undergo standardized evaluations before and after treatment. In addition, two ages have been selected for long-term outcomes: 48 and 60 months. Given that there will be variability in the age of enrollment, some children will receive fewer than 4 evaluations over the course of the study. Post-treatment evaluations ± 3 months of the 48-month evaluation will count as both post-treatment and 48 months; post-treatment evaluations at 57 months or older will count as both post-treatment and 60 months.

### Study measures

The primary clinical outcomes include ASD symptom severity via the BOSCC [32] and cognitive function as measured by the MSEL [33]. For children who reach the ceiling on the MSEL, cognitive function will be assessed via the DAS-II [46], which also measures cognitive function in children. The primary outcomes will examine the group differences in the changes in means from pre- to post-treatment. Of additional interest is the difference between the groups on mean scores at both 48 and 60 months of age. Table 1 provides the measures to be collected and their timing.

**Table 1:** Schedule of measures

Screening measures		Well-child check-ups with pediatric providers				
		<18 months	18 months		24 months	48 months
**Experimental**	M-CHAT-R/F		X		Optional	X
	Provider concerns	Optional	X		Optional	X
	SCQ					X
**Usual care**	M-CHAT-R/F					X
	Provider concerns	Optional	Optional		Optional	X
	SCQ					X
**Evaluation measures**		**Diagnostic**^a^	**Post-treat**^a^		**48 months**	**60 months**
**Parent measures (completed at home)**	CBCL^b^	X	X		X	X
	History forms (family, medical, supplemental)^c^	X	X		X	X
	PDDBI	X	X		X	X
**Evaluation measures**	MSEL	X	X		X	X
	VABS-3	X	X		X	X
	DAS-II					X^d^
	ADOS-2^e^	X			X	X
	DIAL-4					X
	BOSCC	X	X		X	X
	Head circumference	X	X		X	X
	TASI^f^	X	X			
	ADI-R				X	X
	Eye-tracking	X	X		X	X
	Diagnostic Checklist	X	X		X	X
**Treatment measures (at home)**		**Pre-treat**	**During treat**	**Post-treat**^a^	**48 months**	**60 months**
**Parent measures**	PSI-4	X	X^g^		X	X
	FES		X^g^			
**Child measures**	ESDM Checklist	X	X^h^	X		
	CPP	X		X	X	X

*M-CHAT-R/F*, Modified Checklist for Autism in Toddlers, Revised, with Follow-Up; *SCQ*, Social Communication Questionnaire; *CBCL*, Child Behavior Checklist; *PDDBI*, Pervasive Developmental Disorder Behavior Inventory; *MSEL*, Mullen Scales of Early Learning; *Vineland-3*, Vineland Adaptive Behavior Scales, 3rd Edition; *DAS-II*, Differential Abilities Scale, 2nd Edition; *ADOS-2*, Autism Diagnostic Observation Schedule, 2nd Edition; *DIAL-4*, Developmental Indicators for Assessment of Learning, 4th Edition; *BOSCC*, Brief Observation of Social Communication Change; *TASI*, Toddler ASD Symptom Inventory; *ADI-R*, Autism Diagnostic Interview, Revised; *PSI-4*, Parent Stress Index, 4th Edition; *FES*, Family Empowerment Scale; *ESDM*, Early Start Denver Model; *CPP*, Communication Play Protocol

^a^Diagnostic: initial evaluation; post-treatment: evaluation following 12 months of treatment

^b^18 months and older

^c^A re-evaluation history form may be utilized at later evaluations

^d^Use instead of Mullen if skill level at ceiling on Mullen

^e^The appropriate module will be selected based upon the age of the child and verbal ability—toddler module (30 months or less with no phrase speech), module 1 (31 months or more with no phrase speech), and module 2 (phrase speech)

^f^TASI omitted if child older than 36 months

^g^Administered at month 9 of treatment

^h^Administered quarterly during treatment

#### BOSCC

The BOSCC is a measure of global change in autism symptoms in young children with ASD. The BOSCC protocol includes a video-recorded observation of natural interaction between adult and child playing with a pre-defined set of toys and a standardized system to score behaviors related to social communication, play and engagement with objects, stereotypical behaviors, repetitive interests, body mannerisms, and self-injurious behaviors. The BOSCC is administered by a clinician who is blinded to the experimental or usual care group, or the child’s caregiver, and is captured on video. Videos are reviewed, and the BOSCC is scored by trained coders who are blind to the child’s intervention group, age, and the time point of data collection. The BOSCC will be collected prior to treatment, at the conclusion of treatment, at 48 months of age, and at 60 months of age.

#### MSEL

The MSEL is a standardized assessment of cognition widely used in early intervention trials. The four domains assess verbal (expressive language, receptive language) and non-verbal (visual reception, fine motor) cognition and are combined into an early learning composite. The MSEL is administered by a clinician at the start of the evaluation. The MSEL will be administered prior to treatment, at the conclusion of treatment, at 48 months of age, and at 60 months of age.

#### DAS-II

The DAS-II assesses the cognitive function of children as young as 2.5 years and is administered by a trained clinician. The DAS-II will be administered only when a child has reached the maximum score on one or more domains of the MSEL, which we anticipate will only occur during some participants’ 60-month evaluations.

Secondary clinical outcomes include measures of adaptive functioning, ASD symptoms, and kindergarten readiness. Adaptive functioning is measured by the VABS-3 [36], and ASD symptoms are reported with the PDDBI [35]. Both instruments are parent questionnaires to be completed at each evaluation, although the primary comparison of mean values will assess the changes from pre- to post-treatment. Children also will complete the ADOS-2 [34], which measures ASD symptom severity, at each evaluation, and mean values will also be compared between pre- and post-treatment visits. At the 60-month evaluation, children will complete the DIAL-4 [37] to measure readiness for kindergarten, the and mean values will be compared between the two groups.

The secondary outcomes assessing the impact of the intervention on pediatric providers will include descriptive data such as the number of children referred for ASD evaluation, age of children at ASD referral, and physician attitudes and beliefs about screening, as measured by the Provider Belief Survey, adapted from Sices and colleagues [47]. The mean number of children referred and the age of children at referral will be compared between the groups. Physician attitudes and beliefs will be measured at baseline and again at 48 months; the mean changes will be compared between the two groups.

Secondary measures assessing parent outcomes include stress and empowerment. Stress is measured by the Parent Stress Index, 4th Edition [48], and empowerment by the Family Empowerment Scale [49]. These measures will be collected 9 months into treatment, to avoid confounding of change in stress or empowerment as parents transition from treatment delivered by the study team to community or school-based early intervention, and the mean values will be compared between the two groups.

Several approaches are used for exploratory outcomes. These exploratory measures are aimed at assessing treatment-related changes in processes that are targeted by treatment and are not captured by standardized tests. First, a novel battery of assessments based on eye-tracking studies by Vivanti and colleagues [50–52] will measure the treatment changes in social orienting, social cognition, and social motivation. Social orienting will be measured using a preferential looking paradigm developed by Vivanti et al. [52] designed to examine the visual engagement in response to social and non-social stimuli. Social cognition will be measured using a gaze following task [52] that measures participants’ gaze towards the target of an actor’s gaze. An additional measure of social cognition involves a novel eye-tracking paradigm based on the work of Hamlin et al. [53]. This paradigm uses predictive gaze to measure whether participants attribute specific intentions to moving shapes that are shown across different social scenarios.

Social motivation will be measured through the analysis of attentional and emotional engagement in response to (a) social versus non-social rewarding stimuli (e.g., bubbles versus a smiling face) and (b) emotionally engaging versus emotionally neutral stimuli. Following Vivanti et al. [38], participants’ visual attention and changes in pupil diameter (an index of emotional reactivity) are measured through the eye-tracker during the observation of stimuli that vary across the social/nonsocial dimension and emotional valence. As typically developing children experience social versus nonsocial and positive affect versus neutral affect as socially rewarding, increased attentional and emotional responsivity to positive versus neutral affect and social versus non-social rewards is used as an index of social motivation.

Importantly, eye-tracking methods involve several benefits compared to standardized assessments, as they do not rely on the understanding of verbal instructions or previous knowledge and education. Thus, as documented in studies cited above, they are applicable to all children with ASD, regardless of age and symptom severity. Eye-tracking will be performed prior to treatment, at the conclusion of treatment, at 48 months of age, and at 60 months of age, and changes from baseline to each of the time points will be compared between the groups. As these measures are exploratory, determination as to how they will be characterized (e.g., means, medians, counts) will be determined after inspection of the distributions of the data.

Finally, the quality of parent-child interaction will be measured using the Joint Engagement Rating Inventory [54], applied to video-recorded sessions collected prior to treatment, post-treatment, at 48 months, and at 60 months, and the mean changes from baseline will be compared between the groups. This 15-min play session uses the Communication Play Protocol [55] to guide 5-min semi-naturalistic play between the parent and child with three sets of toys to encourage shared exploration of toys within a container, turn taking, and asking for help. At each visit, the order of the Communication Play Protocol activities is randomized, and different sets of toys are used at each time point.

Further, the head circumference will be measured at each evaluation using a tape measure. Two measurements will be taken at each time point, and the larger of the two will be recorded (Amaral, personal communication).

### Quality control

Quality control is assessed in multiple ways in the study. First, pediatric practices are audited randomly on a 3-month cycle to ascertain the number of target well-visits they are billing for. This number is then compared to the number of children screened (for experimental practices). Those who are screening fewer than 80% of children seen in the practice are retrained in study procedures and are then audited the following month. If the proportion of children screened remains below 80%, additional discussions will occur to address the ongoing barriers to high-fidelity screening. After the 18-month screening is completed, we then switch to auditing 48-month well-visits in the same fashion.

A fidelity monitor is employed to assess adherence to the treatment protocol within and across sites. The fidelity monitor, who is a certified ESDM trainer, will randomly select 1 therapy session per therapist per month to be recorded and will review the videos to code pre-specified actions and activities that should or should not be occurring using the ESDM fidelity checklist [31]. Previous research has documented the inter-rater reliability of the ESDM Curriculum Checklist and its utility in clinical trials involving the ESDM [56]. In the case that clinicians are deviating from the protocol (operationalized as fidelity score < 80%) in one or more of the activities recorded in the randomly selected videos, corrective action will be taken based on detailed feedback from the fidelity monitor, in the form of retraining. Another video from the same clinician is subsequently submitted so that the fidelity monitor can ascertain whether treatment implementation is currently at fidelity, or further action is needed. The fidelity monitor will also consider drift in the administration of the treatment over time, as well as differences across sites. In all cases, retraining will be implemented to ensure the standard delivery of treatment across all children.

Data are entered into the web-based data entry system, developed through collaboration between the Clinical Coordinating Center and the Data Coordinating Center. Quality checks are implemented directly into the data entry system to allow real-time validation of incomplete or inconsistent data or out-of-range values. Further, the data entry system allows for double data entry, with a real-time resolution of inconsistent values. All data edits or changes are tracked through the auditing function in the data entry system.

### Participant discontinuation

All attempts will be made to retain participants once they are enrolled. Families receive $50 compensation for each evaluation, and the treatment offered as part of the study is highly desirable to families given that it is intensive, evidence-based early intervention provided by trained therapists. Additional efforts to retain participants include adjusting treatment hours according to the family’s needs and offering alternative locations for treatment, including the child’s home, childcare center, or the university clinic. Should a participant discontinue treatment, we will make every effort to engage them for their post-treatment, 48-month, and 60-month data. Should a participant withdraw from the study completely, we will collect whatever data we can at the time of withdrawal.

### Safety monitoring

The study is monitored by an external Data and Safety Monitoring Board (DSMB), comprising 5 members, including a parent advocate, a pediatric provider, an ethics expert, a biostatistician with experience working with ASD data, and an early intervention expert. The DSMB meets annually and is responsible for ensuring that the study is progressing safely and effectively; DSMB minutes are forwarded to the NIH for reference. No interim efficacy or futility analyses are planned. No major safety outcomes are anticipated nor are any adverse events expected. Any observed adverse events will be classified by their relationship to treatment, will be shared with the DSMB during regular meetings, and will be reported in the final publication. The DSMB Charter is included as an additional file.

### Power calculations

Because there is limited literature describing the differences across age ranges in the effect of early intensive behavioral intervention, effect sizes for the power calculation for the primary analysis were drawn from the literature examining the changes in the outcomes after early intensive behavioral interventions within age ranges. Data for the BOSCC were drawn from Grzadinski et al. [32] and data for the MSEL were drawn from Dawson et al. [57]. Assuming an average of 2 children per practice, and 8 practices per site (48 children per group total), we calculated the power for each of the primary outcomes over a range of intraclass correlations (ICCs), assuming a type I error of 0.05, and a standardized change of either 0.60 or 0.67, with mixed model analyses. Table 2 provides the power for these combinations of parameters. Based on these calculations, we are sufficiently powered to detect standardized changes of this magnitude given the ICC is in the expected range. Power calculations were performed using the GLIMPSE software tool [58] (http://samplesizeshop.org/).

**Table 2:** Power to detect differences in the means across a range of ICCs, for primary outcomes (assuming *n*=96)

Standardize change of 0.67 SDs		Standardized change of 0.60 SDs	
ICC	Power	ICC	Power
0.01	87%	0.01	84%
0.1	86%	0.1	86%
0.2	83%	0.2	76%
0.25	82%	0.25	74%

### Data collection, management, and analysis

Data are collected either via caretaker direct entry into the parent portal of the web-based data entry system or via clinical capture on paper forms and then entered into the web-based data entry system by the site coordinators. Data collected directly from caretakers include informed consent (administered by the site coordinator), demographic data, contact information, M-CHAT-R/F and SCQ screeners, child health-history data, and parent report questionnaires. Pediatric providers indicate ASD concerns via the portal, after which they can see screen results, or by fax to research coordinators. All other data are collected by the clinical teams and subsequently entered electronically. In both cases, validation of data is done in real time, as described above (see the “Quality control” section).

Data entered via the data entry system are stored in a secure location at the Data Coordinating Center and are backed up nightly. Personnel have access to study ID-referenced data only via a secure log-in, except for clinical coordinators who can access personal identifying data in order to communicate with the study participants directly. All changes to the data entry system undergo extensive testing prior to implementation, governed by standard operating procedures. Data exports are available through the data entry system to aid researchers in the preparation of study reports and twice-annual data distribution through the National Data for Autism Research (NDAR) system, as required by the funding agency, and are also governed through the secure log-in to limit data access to those with specific credentials.

Details of the data analysis can be found in the statistical analysis plan. All analyses will be performed under the modified intention to treat principle, unless otherwise stated. That is, all children who initiate treatment will be included in the analyses, regardless of the amount of treatment they receive; however, children of parents who decline treatment are excluded. The primary analysis will address the question of whether children in the experimental group will have a greater magnitude of gains in outcomes compared to children in the usual care group with respect to symptom severity (measured by the BOSCC) and cognitive function (measured by the MSEL or DAS-II). For each outcome, linear mixed models will be fitted to determine whether differences exist between the two groups, allowing for random effects for site, clustering by practice (nested within site), and controlling for the stratification variables (site and size of practice). We will examine several potential covariance structures that make sense for the structure of the data (e.g., unstructured, banded) and will choose based on the Bayesian Information Criteria (BIC). Mixed models are unbiased under the assumption of data missing at random when likelihood estimation is used [59]; thus, we will examine the baseline characteristics of those missing data to those with complete data to determine whether there appears to be any patterns in the missingness. Should there be a large portion of missing data that we suspect are missing not at random, we will utilize multiple imputations to fill in missing values.

Analyses of secondary and exploratory outcomes will be similar to the primary analyses, utilizing mixed models to account for clustering by practice within site and controlling for site and size of practice. We will similarly examine several potential covariance structures and choose based on the BIC. We consider these analyses to be hypothesis-generating, thus will not adjust our results for multiple outcomes.

To assess the difference in the proportion of children referred for evaluation by 48 months between the two groups, we will simply employ an independent test of proportions. We will secondarily fit generalized linear mixed models to compare the proportion referred while accounting for clustering due to practice within the site. To determine whether there is a difference in age at diagnosis between the two groups, we will compare the averages of ages. We will further fit a mixed model to compare the average ages to account for clustering by practice. Physician attitudes and beliefs, measured at baseline and 6-months post-practice launch, will be the sum of 3 key items, with total scores ranging from 5 to 15. We will use linear mixed models, as described above, to analyze this outcome. Should the distribution of the scores not be normal, we will make appropriate transformations, so that the model assumptions are met.

Parent measures will be analyzed using similar approaches to those described above (e.g., linear mixed models, with appropriate transformations if necessary). We will fit a single model to assess differences at 9 months post-treatment onset and a separate model that includes assessments from 60 months of age as well, in order to determine if there are differences at those ages, as well as a trend over time. We will examine the interaction between group (experimental versus usual care) and time and then will examine appropriate contrasts to assess the 60-month time point.

For the third aim, we are interested in whether baseline symptom severity, cognitive functioning, and socioeconomic status moderate the association between the intervention and our outcomes. In order to assess this, we will again fit linear mixed models accounting for clustering by practice within site and including the interaction term between each of these baseline factors and intervention. Because we will likely be underpowered to detect a significant interaction given our sample size, we will fit stratified models and then fit 10,000 bootstrapped samples to estimate the differences in the parameters measuring the association with intervention between the two models and test whether they are statistically significant.

Finally, we will perform pre-specified sensitivity analyses, including the exclusion of children with genetic or metabolic conditions, and assessment of the actual amount of treatment received.

### Dissemination

The results will be disseminated widely through peer-reviewed publications, as well as through presentations at national and international meetings. The publication and presentation policy will guide the study team on issues related to data access, authorship, and other data use concerns.

## Discussion

This paper discusses the study protocol for the Connecting the Dots study, an Autism Center of Excellence Network, with a primary goal of demonstrating differences in short- and long-term outcomes among children diagnosed with ASD through universal, standardized, high-fidelity toddler screening, as compared to usual care. This research will fill a knowledge gap regarding the benefits of universal early, standardized, high-fidelity screening for ASD. Providing evidence of the benefits of universal toddler screening is essential to developing broad recommendations for implementation, according to a recent USPSTF paper (Siu et al. [23]).

The implications of this study for theory, policy, and practice are highly significant. Recent research has shown that up to 70% of children with ASD experience a delay in access to evidence-based intervention, and children that experienced less delay and started treatment at a younger age had better educational outcomes [13]. By rigorously testing the effects of toddler screening, and therefore the age of diagnosis and treatment onset, results will provide insight on the framework that posits that “experience-expectant” plasticity during early development allows for a deeper impact of treatment on the developing brain [5, 60, 61]. If our predictions are supported, the findings will encourage large-scale implementation of toddler screening protocols and provide evidence for the next USPSTF recommendation on universal ASD screening for toddlers. This, in turn, will result in (1) reducing and equalizing the age of ASD detection and treatment across diverse socioeconomic and ethnic backgrounds, (2) optimizing the cost-effectiveness of existing treatments, and (3) encouraging efforts to provide timely early treatment programs in the community. By understanding the impact of the intervention on physician attitudes and on parent empowerment and stress, and by identifying moderators of the relationship between toddler screening and ASD outcomes, recommendations can be made for community implementation and maintenance of screening. This has the potential to optimize outcomes, mitigate the impacts associated with ASD symptoms on the individual or family, reduce care costs, and improve well-being and productivity of individuals with ASD.

The trial does have limitations. First, although our goal is for all eligible children to complete screening, despite our best efforts, it is difficult to ensure that there will no bias in who the pediatric providers choose to encourage to complete the screening, as is shown in other recent studies (e.g., [20]). Further, the results of the trial are reliant on the therapy being implemented in the same fashion across therapists and sites. We will utilize fidelity checks to ensure that clinicians are adhering to the ESDM manual and that there is no drift within or between sites, but this may not completely mitigate the problem. Finally, an important consideration is the relationship between the age of detection and the severity of symptoms and cognitive impairment (our primary outcomes). It is possible that in both groups, more severe cases of ASD will be detected earlier, and our approach to evaluating differences between the groups may be limited in our ability to disentangle these variables. Regardless, we believe that the data we collect will help inform the standard practice for universal screening for ASD.

In order to ensure the best outcomes for children with ASD and their families, it is important to understand the impact of screening and thus the possibility of intervening earlier in the course of the disorder. This trial will help further understand the benefits and drawbacks of standardized, high-fidelity universal screening and should provide evidence as to whether new guidelines are warranted.

### Trial status

This is based on version 2 (dated 5/21/2019) of the protocol. Changes to the protocol will be communicated during monthly cross-site meetings and will be reported to the DSMB at regularly scheduled meetings. Recruitment began on May 31, 2018, and we anticipate that recruitment will end on April 1, 2022.

## Supplementary Information

**Additional file 1.** SPIRIT Checklist.

**Additional file 2.** Statistical Analysis Plan.

**Additional file 3.** DSMB Charter.

**Additional file 4.** Informed Consent Document.

## Data Availability

All findings will be provided by the principal investigator via email; de-identified participant-level data will be available through the NIMH Data Archive (https://nda.nih.gov/). The statistical code corresponding to the analytic results will be available by request from the principal investigator. Public and scientific queries should be directed to the study principal investigator, Dr. Diana Robins: 3020 Market St., Suite 560 Philadelphia, PA 19104-3734 (215) 571-3439 drobins@drexel.edu Scientific queries can also be directed to dotsDCC@drexel.edu.

## Abbreviations

AAP: American Academy of Pediatrics
ADI-R: Autism Diagnostic Interview, Revised
ADOS-2: Autism Diagnostic Observation Schedule-2
ASD: Autism spectrum disorder
BIC: Bayesian Information Criteria
BOSCC: Brief Observation of Social Communication Change
CPP: Communication Play Protocol
DAS-II: Differential Abilities Scale-II
DIAL-4: Developmental Indicators for the Assessment of Learning, Fourth Edition
ESDM: Early Start Denver Model
FES: Family Empowerment Scale
M-CHAT: Modified Checklist for Autism in Toddlers
M-CHAT-R/F: Modified Checklist for Autism in Toddlers, Revised, with Follow-up
MSEL: Mullen Scales of Early Learning
PDDBI: Pervasive Developmental Disorder Behavior Inventory
PSI-4: Parent Stress Index, 4th Edition
RCT: Randomized controlled trials
PPV: Positive predictive value
SCQ: Social Communication Questionnaire
TASI: Toddler ASD Symptom Inventory
USPSTF: United States Preventive Services Task Force

## References

[1] Maenner MJ, Shaw KA, Baio J, Washington A, Patrick M, DiRienzo M (2020). Prevalence of autism spectrum disorder among children aged 8 years - Autism and Developmental Disabilities Monitoring Network, 11 sites, United States, 2016. Morbidity Mortality Weekly Report Surveillance Summaries.

[2] Cakir J, Frye RE, Walker SJ (2020). The lifetime social cost of autism: 1990–2029. Res Autism Spectrum Disord..

[3] Cidav Z, Munson J, Estes A, Dawson G, Rogers S, Mandell D (2017). Cost offset associated with Early Start Denver Model for children with autism. J Am Acad Child Adolesc Psychiatry..

[4] Fuller EA, Kaiser AP (2020). The effects of early intervention on social communication outcomes for children with autism spectrum disorder: a meta-analysis. J Autism Dev Disord..

[5] Landa RJ (2018). Efficacy of early interventions for infants and young children with, and at risk for, autism spectrum disorders. Int Rev Psychiatry..

[6] Smith T, Klorman R, Mruzek DW (2015). Predicting outcome of community-based early intensive behavioral intervention for children with autism. J Abnorm Child Psychol..

[7] Schreibman L, Dawson G, Stahmer AC, Landa R, Rogers SJ, McGee GG (2015). Naturalistic Developmental Behavioral Interventions: empirically validated treatments for autism spectrum disorder. J Autism Dev Disord..

[8] Feuerstein JL, Landa RJ (2020). Implementation of Early Achievements for Childcare Providers: a cluster-randomized controlled trial. Early Childhood Res Quarterly..

[9] Fuller EA, Oliver K, Vejnoska SF, Rogers SJ. The effects of the Early Start Denver Model for children with autism spectrum disorder: a meta-analysis. Brain Sci. 2020;10(6):368. 10.3390/brainsci10060368 PMC7349854 32545615

[10] Tiede G, Walton KM (2019). Meta-analysis of Naturalistic Developmental Behavioral Interventions for young children with autism spectrum disorder. Autism..

[11] Estes A, Munson J, Rogers SJ, Greenson J, Winter J, Dawson G (2015). Long-term outcomes of early intervention in 6-year-old children with autism spectrum disorder. J Am Acad Child Adolesc Psychiatry..

[12] Pickles A, Le Couteur A, Leadbitter K, Salomone E, Cole-Fletcher R, Tobin H (2016). Parent-mediated social communication therapy for young children with autism (PACT): long-term follow-up of a randomised controlled trial. Lancet..

[13] Dimian AF, Symons FJ, Wolff JJ. Delay to Early Intensive Behavioral Intervention and Educational Outcomes for a Medicaid-Enrolled Cohort of Children with Autism. J Autism Dev Disord. 2021;51(4):1054–66. 10.1007/s10803-020-04586-1 PMC12599662 32642958

[14] MacDonald R, Parry-Cruwys D, Dupere S, Ahearn W (2014). Assessing progress and outcome of early intensive behavioral intervention for toddlers with autism. Res Dev Disabil..

[15] Vivanti G, Dissanayake C (2016). Outcome for children receiving the Early Start Denver Model before and after 48 months. J Autism Dev Disord..

[16] Vivanti G, Dissanayake C, Duncan E, Feary J, Capes K, Upson S (2019). Outcomes of children receiving Group-Early Start Denver Model in an inclusive versus autism-specific setting: a pilot randomized controlled trial. Autism..

[17] Robins DL, Fein D, Barton M (1999). The Modified Checklist for Autism in Toddlers (M-CHAT).

[18] Robins DL, Fein D, Barton M (2009). The Modified Checklist for Autism in Toddlers, Revised, with Follow-up (M-CHAT-R/F).

[19] Robins DL, Casagrande K, Barton M, Chen CM, Dumont-Mathieu T, Fein D (2014). Validation of the Modified Checklist for Autism in Toddlers, Revised with Follow-up (M-CHAT-R/F). Pediatrics..

[20] Guthrie W, Wallis K, Bennett A, Brooks E, Dudley J, Gerdes M, et al. Accuracy of Autism Screening in a Large Pediatric Network. Pediatrics. 2019;144(4):e20183963. 10.1542/peds.2018-3963 31562252

[21] Stenberg N, Bresnahan M, Gunnes N, Hirtz D, Hornig M, Lie KK (2014). Identifying children with autism spectrum disorder at 18 months in a general population sample. Paediatric Perinatal Epidemiol..

[22] Herlihy LE, Brooks B, Dumont-Mathieu T, Barton ML, Fein D, Chen CM (2014). Standardized screening facilitates timely diagnosis of autism spectrum disorders in a diverse sample of low-risk toddlers. J Dev Behav Pediatr..

[23] Siu AL, Bibbins-Domingo K, Grossman DC, Baumann LC, Davidson KW, Ebell M (2016). Screening for autism spectrum disorder in young children: US Preventive Services Task Force Recommendation Statement. JAMA.

[24] Johnson CP, Myers SM (2007). Identification and evaluation of children with autism spectrum disorders. Pediatrics..

[25] Hyman SL, Levy SE, Myers SM. Identification, evaluation, and management of children with autism spectrum disorder. Pediatrics. 2020;145(1):e20193447. 10.1542/peds.2019-3447 31843864

[26] Dosreis S, Weiner CL, Johnson L, Newschaffer CJ (2006). Autism spectrum disorder screening and management practices among general pediatric providers. J Dev Behav Pediatr..

[27] Arunyanart W, Fenick A, Ukritchon S, Imjaijitt W, Northrup V, Weitzman C (2012). Developmental and autism screening: a survey across six states. Infants Young Children..

[28] Self TL, Parham DF, Rajagopalan J (2015). Autism spectrum disorder early screening practices: a survey of physicians. Commun Disord Quarterly..

[29] Sánchez-García AB, Galindo-Villardón P, Nieto-Librero AB, Martín-Rodero H, Robins DL (2019). Toddler screening for autism spectrum disorder: a meta-analysis of diagnostic accuracy. J Autism Dev Disord..

[30] Institute of Medicine Committee on Quality of Health Care in A (2001). Crossing the quality chasm: a new health system for the 21st century.

[31] Rogers SJ, Dawson G (2009). Early Start Denver Model curriculum checklist for young children with autism.

[32] Grzadzinski R, Carr T, Colombi C, McGuire K, Dufek S, Pickles A (2016). Measuring changes in social communication behaviors: preliminary development of the Brief Observation of Social Communication Change (BOSCC). J Autism Dev Disord..

[33] Mullen EM (1995). Mullen scales of early learning.

[34] Lord C, Rutter M, DiLavore P, Risi S, Gotham K, Bishop S (2012). Autism diagnostic observation schedule, (ADOS-2) modules 1-4.

[35] Cohen IL, Schmidt-Lackner S, Romanczyk R, Sudhalter V (2003). The PDD Behavior Inventory: a rating scale for assessing response to intervention in children with pervasive developmental disorder. J Autism Dev Disord..

[36] Sparrow S, Cicchetti D, Saulnier C (2016). Vineland Adaptive Behavior Scales–third edition (Vineland-3).

[37] Mardell C, Goldenberg D (2010). DIAL-4: developmental indicators for the assessment of learning.

[38] Vivanti G, Hocking DR, Fanning P, Dissanayake C (2016). Social affiliation motives modulate spontaneous learning in Williams syndrome but not in autism. Mol Autism..

[39] Adamson LB, Bakeman R, Deckner DF, Nelson PB (2012). Rating parent-child interactions: joint engagement, communication dynamics, and shared topics in autism, Down syndrome, and typical development. J Autism Dev Disord..

[40] Suma K, Adamson LB, Bakeman R, Robins DL, Abrams DN (2016). After early autism diagnosis: changes in intervention and parent-child interaction. J Autism Dev Disord..

[41] Barton ML, Boorstein H, Dumont-Mathieu T, Herlihy LE, Fein D (2012). Toddler ASD Symptom Interview (TASI).

[42] Lord C, Rutter M, Le Couteur A (1994). Autism Diagnostic Interview-Revised: a revised version of a diagnostic interview for caregivers of individuals with possible pervasive developmental disorders. J Autism Dev Disord..

[43] Rutter M, Bailey A, Lord C, Berument S (2003). Social communication questionnaire (SCQ).

[44] Rogers SJ, Dawson G (2010). Early Start Denver Model for young children with autism: promoting language, learning, and engagement.

[45] Rogers SJ, Dawson G, Press G (2010). Early Start Denver Model curriculum checklist for young children with autism.

[46] Elliott C (2007). Differential Ability Scales®-IIDAS-II.

[47] Sices L, Feudtner C, McLaughlin J, Drotar D, Williams M (2003). How do primary care physicians identify young children with developmental delays? A national survey. J Dev Behav Pediatr..

[48] Abidin RR (2012). Parenting Stress Index 4th Edition-Professional Manual.

[49] Koren PE, DeChillo N, Friesen BJ (1992). Measuring empowerment in families whose children have emotional disabilities: a brief questionnaire. Rehabil Psychol..

[50] Nuske HJ, Vivanti G, Dissanayake C (2015). No evidence of emotional dysregulation or aversion to mutual gaze in preschoolers with autism spectrum disorder: an eye-tracking pupillometry study. J Autism Dev Disord..

[51] Nuske HJ, Vivanti G, Dissanayake C (2016). Others’ emotions teach, but not in autism: an eye-tracking pupillometry study. Mol Autism..

[52] Vivanti G, Fanning PAJ, Hocking DR, Sievers S, Dissanayake C (2017). Social attention, joint attention and sustained attention in autism spectrum disorder and Williams syndrome: convergences and divergences. J Autism Dev Disord..

[53] Hamlin JK, Wynn K, Bloom P (2007). Social evaluation by preverbal infants. Nature..

[54] Adamson LB, Bakeman R, Suma K (2016). The Joint Engagement Rating Inventory (JERI). Technical report 25.

[55] Adamson LB, Bakeman R (2016). The communication play protocol: capturing variations in language development. Perspect ASHA Spec Interest Groups..

[56] Vismara LA, Young GS, Rogers SJ (2012). Telehealth for expanding the reach of early autism training to parents. Autism Res Treat..

[57] Dawson G, Rogers S, Munson J, Smith M, Winter J, Greenson J (2010). Randomized, controlled trial of an intervention for toddlers with autism: the Early Start Denver Model. Pediatrics..

[58] Kreidler SM, Muller KE, Grunwald GK, Ringham BM, Coker-Dukowitz ZT, Sakhadeo UR, et al. GLIMMPSE: Online Power Computation for Linear Models with and without a Baseline Covariate. J Stat Softw. 2013;54(10):i10. 10.18637/jss.v054.i10 PMC3882200 24403868

[59] Little RJ, Raghunathan T (1999). On summary measures analysis of the linear mixed effects model for repeated measures when data are not missing completely at random. Stat Med..

[60] Sullivan K, Stone WL, Dawson G (2014). Potential neural mechanisms underlying the effectiveness of early intervention for children with autism spectrum disorder. Res Dev Disabil..

[61] Vivanti G, Zhong N (2020). Naturalistic Developmental Behavioral Interventions for children with autism spectrum disorder. Clinical guide to early interventions for children with autism.

