# Supplementary material for: Connecting the Dots: a cluster-randomized clinical trial integrating standardized autism spectrum disorders screening, high-quality treatment, and long-term outcomes
Source: Trials. 2021 May 2;22:319. doi: 10.1186/s13063-021-05286-6 (PMC8091523; doi:10.1186/s13063-021-05286-6)
Supplement: Supplementary file 2 — Additional file 2. Statistical Analysis Plan. [file 13063_2021_5286_MOESM2_ESM.docx]

Promoting Positive Outcomes for Individuals with ASD: Linking Early Detection, Treatment, and Long-term Outcomes

Statistical Analysis Plan


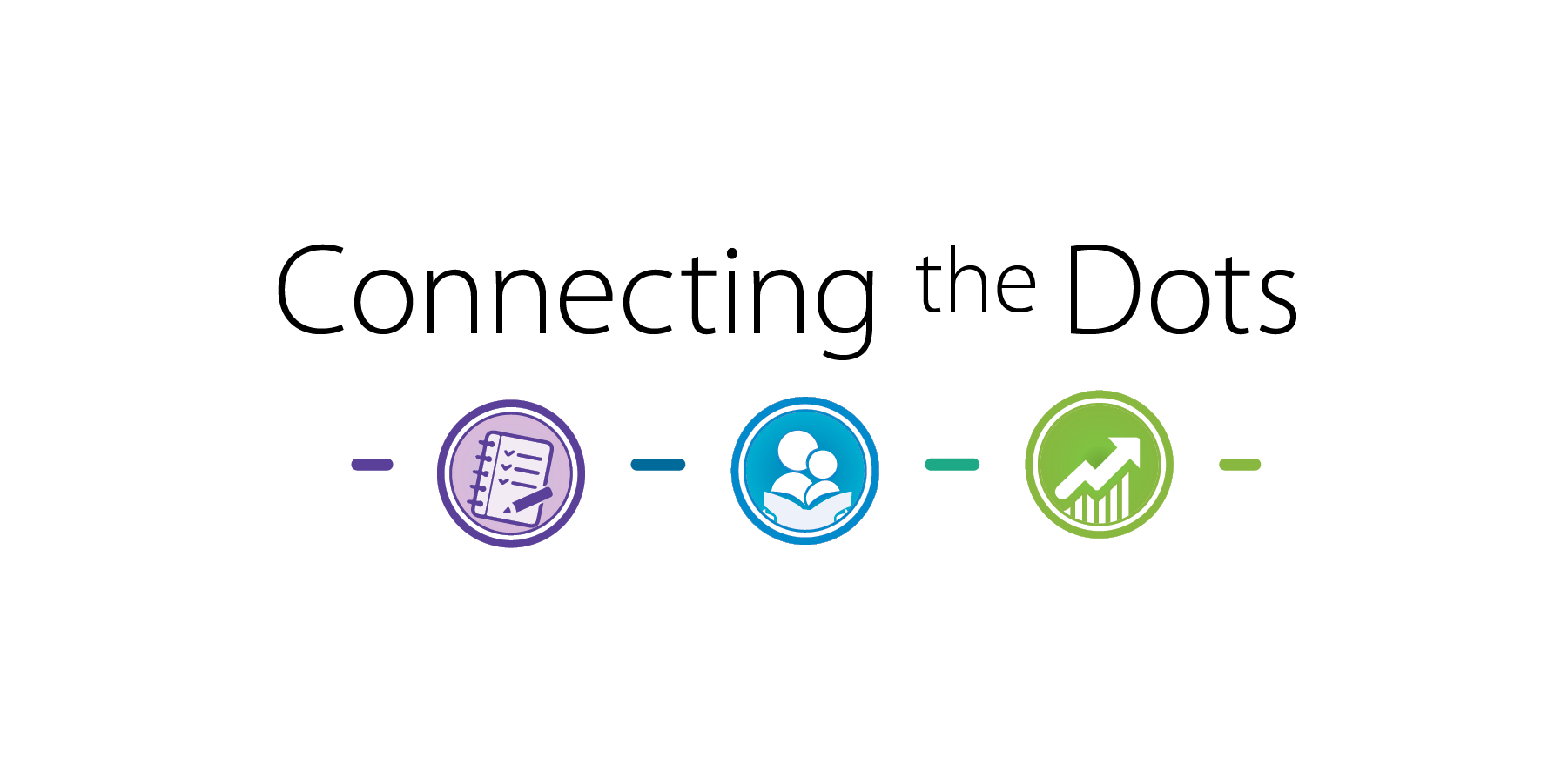


Prepared By:

Connecting the Dots Data Coordinating Center

Leslie McClure, PhD (PI)

Study PI: Diana Robins, PhD

Department of Epidemiology and Biostatistics

Dornsife School of Public Health

Philadelphia, PA

Version 1.1

October 18, 2018

clinicaltrials.gov: NCT03333629

**TABLE OF CONTENTS**

1. Background and Rationale1

Type chapter title (level 2)

Type chapter title (level 3)

2. Objectives2

Type chapter title (level 2)5

Type chapter title (level 3)6

3. Statistical Analyses3

3.1 Aim 13

3.1.1 Primary Analyses3

3.1.2 Power Calculations4

3.1.3 Secondary Analyses4

3.2 Aim 24

3.2.1 Primary Analyses4

3.2.2 Sample Size5

3.3 Aim 35

3.3.1 Primary Analyses5

3.4 Tertiary Analyses5

3.5 Missing Data5

4. References6

**1. Background and Rationale**

Autism Spectrum Disorder (ASD) currently affects up to one in 59 children in the US (CDC, 2017). ASD can result in lifelong disability, and lead to physical, emotional and economic burdens at the individual, family and society level. Overall, annual costs associated with ASD in the US were estimated to be $268 billion for 2015 (Leigh & Du, 2015), exceeding the costs of stroke and hypertension.

**ASD-specific** **treatment** can change life course trajectories of children with ASD, mitigating later adult disability, improving family well-being, and reducing societal costs (Dawson & Burner, 2011; Eikeseth et al., 2015; Volkmar & Reichow, 2014). Treatment effects in a number of RCTs include gains in cognitive, adaptive, and social-communication functioning, both immediately after treatment (Rogers & Talbott, 2016; Zwaigenbaum et al., 2015) and several years after treatment cessation (Estes et al., 2015; Pickles et al., 2016). Furthermore, recent research indicates that **children who begin treatment before their fourth birthday achieve better outcomes** compared to those who delay treatment onset (Anderson et al., 2014; MacDonald et al., 2014; Vivanti et al., 2016).

Figure 1. Schematic illustrating the hypothesised effect of ASD screening


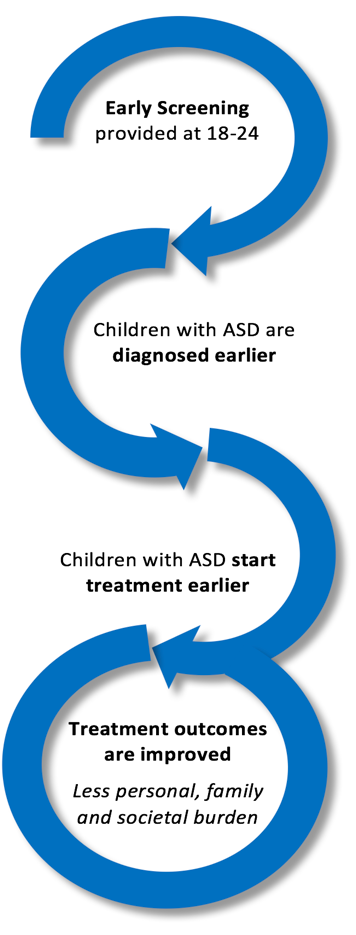


The most widely used ASD screener is the Modified Checklist for Autism in Toddlers (M-CHAT; Robins, Fein, & Barton, 1999), and its revision, the M-CHAT Revised, with Follow-Up (M-CHAT-R/F; Robins, Fein, & Barton, 2009). This two-stage screening tool has 20 yes/no items that parents complete during well-child visits; when children score moderate risk, parents complete 3-7 Follow-Up items to clarify risk status, whereas high-risk children are immediately referred for evaluation. In an unselected sample of more than 16,000 children (mean age=20.9m, SD=3.3) screened in well-child visits (Robins et al., 2014), with multiple strategies to detected missed cases, the M-CHAT-R/F demonstrated adequate psychometrics: sensitivity=.83, specificity=.99, positive predictive value (PPV)=.45, negative predictive value (NPV)=.99. Recent evidence suggests that **implementation of the M-CHAT-R/F, coupled with immediate referrals, can lower the age of diagnosis by two years** (Robins et al., 2014). This finding, along with evidence that starting treatment at a younger age is associated with improved outcomes, suggests that early screening has the potential to improve the lives of individuals with ASD (see Figure 1).

**Direct evidence for this impact of screening on outcomes is still incomplete**. Based on the analysis of the existing literature, the US Preventive Services Task Force (USPSTF) recently indicated that the evidence for ASD screening in the general pediatric population is not sufficient to recommend universal screening (Siu et al., 2016). In particular, they highlighted the lack of **randomized controlled trials (RCTs) testing the outcomes of children detected through screening** **and referred to treatment compared to children detected in other ways**. This project will address this gap in knowledge through **an RCT examining the effects of early, standardized, high-fidelity screening for ASD vs. usual care on short- and long-term outcomes**.

**2. Objectives**

The overall goal of this project is to **examine the effects of evidence-based early screening for ASD on short- and long-term child outcomes compared to outcomes for children receiving usual care**. We will test the hypothesis that children with ASD in the early screening group (high-fidelity early screening) will achieve superior outcomes compared to children receiving usual care, as a result of earlier detection and earlier onset of high-quality treatment. We will test our hypothesis through assessing the following aims:

*Aim 1*. **Compare short- and long-term outcomes** between the experimental (high-fidelity, standardized, universal screening at 18 m) and the usual care groups. Given that the primary difference between groups is the age of detection (due to the training in early, standardized, high-fidelity screening in the early screening group), it is predicted that younger age of screening, and therefore of diagnosis and treatment onset, will result in superior outcomes for children in the experimental group. **(1a) Short-term clinical outcomes** will be measured at the end of 12-months of Early Intensive Behavioural Intervention (EIBI), and **(1b) long-term outcomes** will be measured at 48 and 60 months old. Primary outcomes will be cognitive functioning and ASD symptom severity. Secondary outcomes include adaptive functioning, and at 60 months only, kindergarten readiness. Additional exploratory outcomes will include experimental measures of changes directly relevant to the social reciprocity processes targeted by treatment, including eye-tracking and pupillometry paradigms (Nuske et al., 2016; Vivanti et al., 2016) and parent-child social engagement (Adamson et al., 2012; Suma et al., 2016).

*Aim 2.* **Evaluate the impact of the intervention – delivery of universal, standardized, high-fidelity screening – on physician and practice variables**, including the number of children referred for ASD evaluation, age of children at referral, and physician attitudes and beliefs about screening, as well as **parent variables**, including stress, and empowerment. We expect that in the early intervention group, **(2a)** more children will be identified with ASD prior to the 48 m screen, **(2b)** physicians will have more positive attitudes about screening, **(2c)** parent empowerment will be higher, and **(2d)** parent stress will be lower, compared to the usual care group.

*Aim 3.* **Evaluate moderators of the effect of standardized early screening on short- and long-term outcomes.** We will examine baseline symptom severity, cognitive functioning, and socioeconomic status (SES) as potential moderators. We predict that children with milder symptoms, and greater cognitive ability will benefit more from early screening. Given the equivocal literature regarding SES and early detection, and the heterogeneity of SES in the proposed sample, an exploratory analysis will examine SES as a potential moderator.

**3. Statistical Analyses**

The remainder of this document describes the analysis plan and power calculations for the aims described above. Unless otherwise specified, all analyses will follow the intention to treat principle. Randomization for this study occurs at the practice level, stratified within research site (Drexel. UC Davis, UConn), and stratified by size of practice (as measured by average number of 18 month visits that occur in a typical month, and stratified as small and large with a cut point of 20 visits).

*3.1 Aim 1: Compare short- and long-term outcomes between the experimental and usual care groups.*

*3.1.1 Primary Analyses*

The primary analysis will address the question of whether children in the early intervention group will have better gains in outcomes compared to children in the usual care group with respect to symptom severity (measured by the Brief Observation of Social Communication Change: BOSCC; and Autism Diagnostic Observation Schedule-2: ADOS-2) and cognitive function (measured by the Mullen Scales of Early Learning: MSEL). For each outcome, linear mixed models will be fitted to determine whether differences exist between the two groups, allowing for random effects for site, clustering by practice (nested within site), and controlling for site and size of practice (small vs. large). We will examine several potential covariance structures that make sense for the structure of the data (e.g. unstructured, banded), and will choose based on the Bayesian Information Criteria (BIC).

*3.1.2 Power Calculations*

****Because limited literature describing differences across age ranges in the effect of EIBI exists, effect sizes for the power calculation for the primary analysis were drawn from literature examining changes in outcomes after EIBI within age ranges. Data for the BOSCC were drawn from Grzadinski *et al* (2016) and data for the MSEL were drawn from Dawson *et al* (2010). Assuming an average of 2 children per practice, and 8 practices per site for each group, we calculated power for each of the primary outcomes over a range of intraclass correlations (ICCs), assuming a Type I error of 0.05, and a standardized change of either 0.60 or 0.67, with mixed model analyses. Table 2 provides the power for these combinations of parameters. Power calculations were performed using the GLIMPSE software tool (Kriedler *et al*, 2013: <http://samplesizeshop.org/>).

*3.1.3 Secondary Analyses*

Secondary outcomes include: adaptive functioning, kindergarten readiness, and parent report of ASD symptoms (PDDBI). Analyses of secondary outcomes will be similar to the primary analyses, utilizing mixed models to account for clustering by practice within site, and controlling for site and size of practice. We will similarly examine several potential covariance structures, and choose based on the BIC. We consider these analyses to be hypothesis generating, thus will not adjust our results for multiple outcomes.

*3.2 Aim 2: Evaluate the impact of the intervention on physician and practice variables as well as parent variables.*

*3.2.1 Primary Analyses*

The second aim is concerned with assessing the impact of the intervention on physician and practice measures, as well as parent measures. The primary physician/practice variables of interest include the number of children referred for ASD evaluation, age of children at referral, and physician attitudes and beliefs about screening. To compare the proportion of children referred for ASD evaluation by 48 months between the two groups, we will simply employ an independent test of proportions. We will secondarily fit generalized linear mixed models to compare the proportion referred, while accounting for clustering due to practice within site. To determine whether there is a difference in age between the two groups, we will compare averages of ages. We will further fit a mixed model to compare the average ages to account for clustering by practice. Physician attitudes and beliefs, measured at baseline and 6-months post practice launch, will be the sum of 3 key items, with total scores ranging from 5-15. We will use linear mixed models, as described above, to analyze this outcome. Should the distribution of the scores not be normal, we will make appropriate transformations, in order that the model assumptions are met.

Parent measures include: satisfaction with screening and evaluation, stress, and empowerment, and will be analyzed using similar approaches to those described above (e.g. linear mixed models, with appropriate transformations if necessary). We will fit a single model to assess differences at 9 months post intervention, and a separate model that includes assessments from 60 months of age as well, in order to determine if there are differences at those ages, as well as a trend over time. We will examine the interaction between intervention and time, and then will examine appropriate contrasts to assess the 60 month time-point.

*3.2.2 Sample size*

Based on prior work (Robins et al., 2014), we assume that approximately 86% of the children in the early screening group will be diagnosed prior to 48 months, whereas about 50% of the children in the usual care group will be detected prior to 48 months. Given our sample size and assuming a type I error rate of 0.05, using a test of independent proportions, we have >95% power to detect this difference. For the physician measure (attitudes) Table 2 provides the minimum detectable differences across different values of the ICC for which we have approximately 80% power. Based on previous literature (Eikeseth et al., 2015; Minjarez et al., 2012; Rivard et al., 2014), we predict that parent stress will be lower at both 9 months after EIBI onset, and at the 60 months long-term evaluation in the early screening vs. usual care group; similar to stress, we expect greater empowerment for the early screening vs. usual care group. Table 2 shows the minimum detectable difference in the parent stress and empowerment outcomes that we can expect to detect, using the same approach for power as in Aim 1.

*3.3 Aim 3: Evaluate moderators of the effect of standardized early screening on short- and long-term outcomes.*

*3.3.1 Primary Analyses*

We are interested in whether baseline symptom severity, cognitive functioning, and SES moderate the association between the intervention and our outcomes. In order to assess this, we will again fit linear mixed models accounting for clustering by practice within site, and including the interaction term between each of these baseline factors and intervention. Because we will likely be underpowered to detect a significant interaction given our sample size, we will fit stratified models, and then fit 10,000 bootstrapped samples to estimate the differences in the parameters measuring the association with intervention between the two models and test whether they are statistically significant. For example, we will fit a model for boys and girls, and then compare whether the parameter for intervention differs between the two models.

*3.4 Tertiary Analyses*

Tertiary outcomes include:

- Eye tracker data
- Head Circumference

*3.5 Missing Data*

While we will do our best to ensure that data are as complete as possible, we recognize that missing data are always a concern. Our primary analysis will incorporate only the observed data. Assuming that data are missing at random, and given that we are using mixed models, this should allow our treatment effects to be estimated without bias (although less efficiently). As a sensitivity analysis, we will use multiple imputation to fill in any missing values, and fit the same models.

*3.6 Sensitivity Analyses*

Although we recognize that including siblings in the analysis may induce correlation among the participants, we also recognize that this is unavoidable. Thus, we will do sensitivity analyses in which we randomly select a single child from among siblings in order to ensure that the correlation does not impact our results.

Further, we will do sensitivity analyses in which we exclude children with genetic or metabolic conditions, in order to ensure that these children do not bias our results.

Added October 18, 2019: Additional sensitivity analyses will examine the impact of the “dose” of treatment (average number of hours per week) among those children who are treated. This is similar to an as-treated analysis, as opposed to the planned intention-to-treat.

**4. References**
