# Supplementary material for: Connecting the Dots: a cluster-randomized clinical trial integrating standardized autism spectrum disorders screening, high-quality treatment, and long-term outcomes
Source: Trials. 2021 May 2;22:319. doi: 10.1186/s13063-021-05286-6 (PMC8091523; doi:10.1186/s13063-021-05286-6)
Supplement: Supplementary file 3 — Additional file 3. DSMB Charter. [file 13063_2021_5286_MOESM3_ESM.doc]

# **DSMB Charter Promoting Positive Outcomes for Individuals with ASD: Linking Early Detection, Treatment, and Long-Term Outcomes**

# **Dr. Diana Robins, PI**

# **National Institute of Mental Health: 1R01MH115715-01**


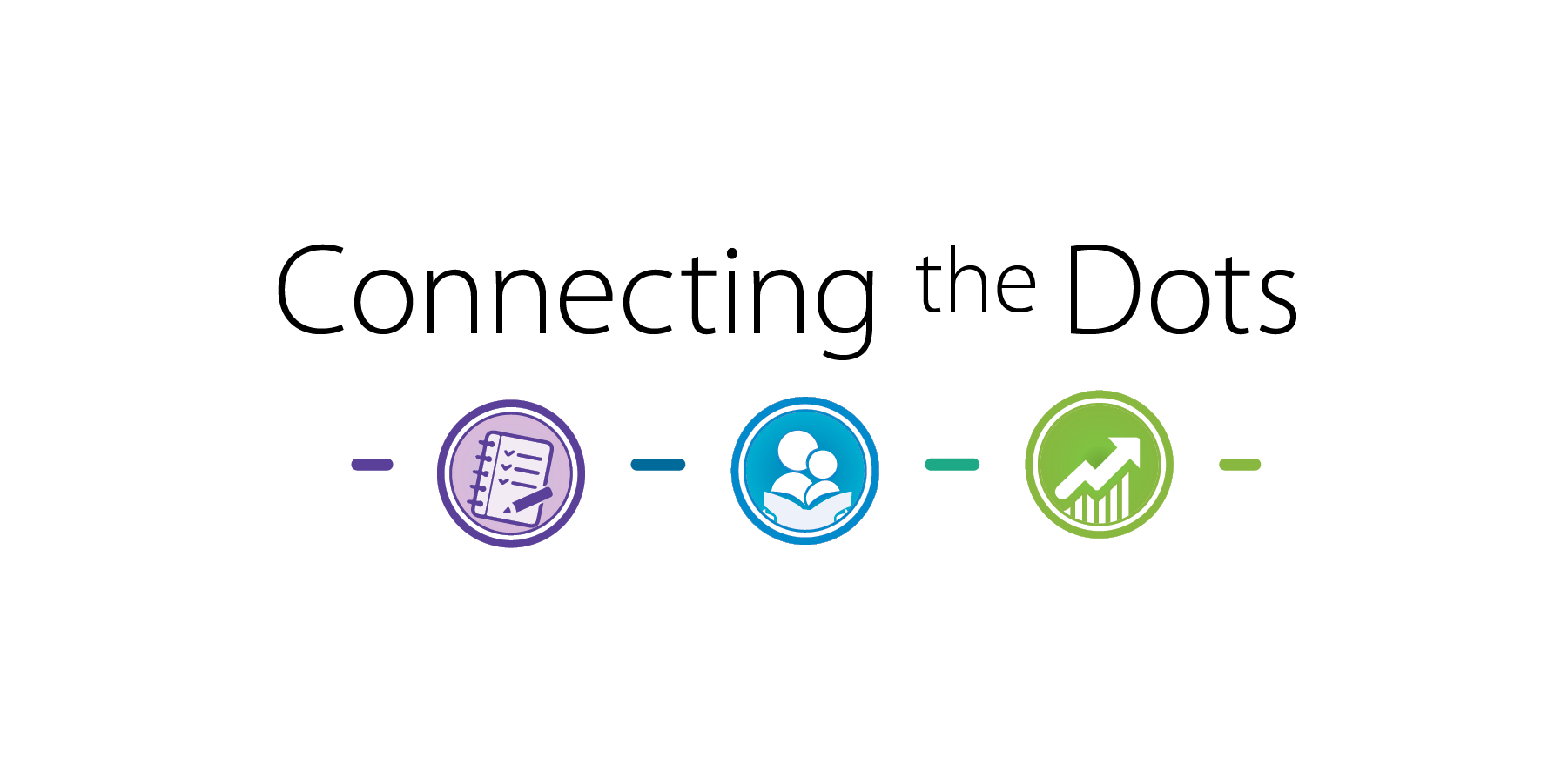

The Data and Safety Monitoring Board (DSMB) will act in an advisory capacity to the National Institute of Mental Health (NIMH) to monitor participant safety, data quality and evaluate the progress of the study. Dr. Diana Robins at Drexel University is conducting the Promoting Positive Outcomes for Individuals with ASD: Linking Early Detection, Treatment, and Long-term Outcome (Connecting the Dots) study under a grant funded by the NIMH.

DSMB Responsibilities

The DSMB responsibilities are to:

- review the study documents and plans for data safety and monitoring;
- evaluate the progress of the trial, including periodic assessments of data quality and timeliness, recruitment, accrual and retention, participant risk versus benefit, performance of the trial sites, and other factors that can affect study outcome;
- consider factors external to the study when relevant information becomes available, such as scientific or therapeutic developments that may have an impact on the safety of the participants or the ethics of the trial;
- review study performance, make recommendations and assist in the resolution of problems reported by the Principal Investigator;
- protect the safety of the study participants;
- make recommendations to the NIMH and the Principal Investigator concerning continuation, termination or other modifications of the trial based on the observed beneficial or adverse effects of the treatment under study;
- assist the NIHM by commenting on any problems with study conduct, enrollment, sample size and/or data collection.

The DSMB will discharge itself from its duties when the last participant completes the study.

Membership

The DSMB comprises 5 members, one of whom is chair, all of whom are voting members of the Board.

Dr. Alycia Halladay, Autism Science Foundation will serve as the parent advocate to the DSMB. She also will serve as the Chairperson of the DSMB and is responsible for overseeing the meetings and developing the agenda in consultation with the Principal Investigator. The Chair is the contact person for the DSMB. Drexel University will provide the logistical management and support of the DSMB.

Dr. Ho Wen Hsu, MD will serve as the Pediatric Provider on the DSMB. Dr. Hsu is a pediatric provider in private practice in Massachusetts and will offer perspective from the provider point of view.

Dr. Erin Paquette, MD, JD, Assistant Professor of Pediatrics, Feinberg School of Medicine, will serve as the ethics expert on the DSMB.

Dr. Elizabeth Stuart, PhD, Professor of Mental Health, Biostatistics, and Health Policy and Management, Associate Dean for Education, Johns Hopkins Bloomberg School of Public Health, will serve as the DSMB statistician.

Ms. Colleen Tessing, MEd, BCBA, from Interactive Kids, will serve as the Early Intervention Provider on the DSMB.

## Board Process

At the first meeting the DSMB will discuss the protocol, and establish guidelines to study monitoring by the Board. The DSMB Chairperson, in consultation with the Principal Investigator, will prepare the agenda to address the review of study materials, statistical analysis plan including interim analysis and mock DSMB report.

Meetings of the DSMB will be held at least one time a year at the call of the Chairperson and / or Principal Investigator, unless deemed necessary by the DSMB.

Meetings are closed to the public because discussions may address confidential participant data. Meetings are attended by the Principal Investigator, the Data Coordinating Center (DCC) Principal Investigator, Site Investigators, and members of the study staff, as determined by the study leadership. Meetings will be convened as conference calls.

**Meeting Format**

DSMB meetings will consist of open and closed sessions (if necessary). Discussion held in all sessions is confidential. The Principal Investigator and key members of the study team attend the **open sessions.** Open session discussion will focus on the conduct and progress of the study, including participant accrual, protocol compliance, and problems encountered. Unblinded data are not presented in the open session.

If a **closed session** is necessary, it will be attended by the DSMB members. The study statistician may be present, at the request of the DSMB. Any data by blinded study group and, as necessary, unblinded data, are presented during the closed session, if at some point in time the DSMB determines that it is necessary to view data by group.

An **executive session** will be attended by voting DSMB members. The executive session will be held to identify and discuss the DSMB’s recommendations to the study staff and NIMH. The study staff may be present, at the request of the DSMB, during the executive session.

Each meeting must include a recommendation to continue or to terminate the study and whether the DSMB has any concerns about study conduct or participant safety. Should the DSMB decide to issue a termination recommendation, the full vote of the DSMB is required. In the event of a split vote, majority vote will rule and a minority report should be appended. The DSMB Chair provides the tiebreaking vote in the event of a 50-50 split vote.

**Meeting Materials**

DSMB report templates will be prepared by the DCC staff, and will be reviewed by the DSMB members at the first meeting. For this study, DSMB reports will typically only contain an Open Session Report, unless otherwise determined by the DSMB.

Format and content of the reports will be finalized and approved at the initial DSMB meeting, although changes throughout the trial may be requested by the Board.

The reports will summarize safety data and describe the status of the study. All meeting materials should be sent to the DSMB at least 7 to 14 days prior to the meeting. Reports will be sent via a password-protected PDF file.

**Open Session Reports:** The open session reports will include administrative reports by site that describe participants screened, evaluated, enrolled in treatment, and completed treatment. Further, early terminations (and reasons) and protocol deviations will be described. Baseline characteristics of the study population in aggregate will be described. Quality control and assurance will be described through tables presenting screening rates by practice (with names redacted so as to not unblind practices), and for those in treatment, proportion of expected visits that are completed. Other general information on study status may also be presented. Listings of adverse events and serious adverse events, as well as any other information requested by the DSMB, may also be in the open session report, but none of the data will be presented by intervention group due to the potential for unblinding. The DSMB may direct additions and other modifications to the reports on a one-time or continuing basis.

**Reports from the DSMB**

A formal report containing the meeting minutes, including recommendations for continuation or modifications of the study will be prepared by the DSMB Chairperson. The draft report will be sent to the DSMB members for review and approval. Once approved by the DSMB members, the DSMB Chairperson will forward the report to the Principal Investigator, who will share it with NIMH staff. It is the responsibility of the Principal Investigator to distribute the DSMB recommendation to all co-investigators and to ensure that copies are submitted to all the IRBs associated with the study.

**Confidentiality**

All materials, discussions and proceedings of the DSMB are completely confidential. Members and other participants in DSMB meetings are expected to maintain confidentiality.
