# Supplementary material for: Connecting the Dots: a cluster-randomized clinical trial integrating standardized autism spectrum disorders screening, high-quality treatment, and long-term outcomes
Source: Trials. 2021 May 2;22:319. doi: 10.1186/s13063-021-05286-6 (PMC8091523; doi:10.1186/s13063-021-05286-6)
Supplement: Supplementary file 4 — Additional file 4. Informed Consent Document. [file 13063_2021_5286_MOESM4_ESM.pdf]

Drexel University
Consent to Take Part in a Research Study
Family Consent Form – Step 2

1. ***Title of research study:*** Connecting the Dots: Early Detection, Diagnosis, and Treatment for Developmental Delays

2. ***Researcher:*** Diana L. Robins, Ph.D.

3. *Why you are being invited to take part in a research study*

We invite you to take part in a research study because you have completed step 1 by filling out a questionnaire at your pediatric provider's office. We are inviting you to participate in the next part of this study because your child may have some risk of developmental delays. Some children qualify to receive help as part of this study. This form will tell you more about this part of the study. You can decide whether you would like to be in the study. In this form, "you" means you and your child.

4. *What you should know about a research study*

- Someone will explain this research study to you.
- Whether or not you take part is up to you.
- You can choose not to take part.
- You can agree to take part now and change your mind later.
- If you decide to not be a part of this research no one will hold it against you.
- Feel free to ask all the questions you want before you decide.

5. *Who can you talk to about this research study?*

If you have questions, concerns, or complaints, or think the research has hurt you, talk to the research team at your university. You can reach Dr. Robins (Drexel University) at 215-571-3439. You can reach Dr. Fein (University of Connecticut) at 860-486-5767. You can reach Dr. Stahmer (University of California, Davis) at (916) 703-0254. You can reach Dr. Dumont-Mathieu (Connecticut Children's Medical Center and The Connecticut Office of Early Childhood) at 860-837-5758. You can reach Lauren Schmock (Abington Hospital- Jefferson Health) at 215-855-8296. You can reach Diane Treadwell-Deering (Nemours/A I Dupont Hospital for Children) at 302-651-4876. You can reach Katharine Hemady (Public Health Management Corporation - PHMC) at 215-731-2127.

This research has been reviewed and approved by an Institutional Review Board (IRB) at Drexel University. An IRB reviews research projects so that steps are taken to protect the rights and welfare of human subjects taking part in research. The IRBs at the University of Connecticut, Connecticut Children's Medical Center, and the University of California, Davis, have agreed to rely on Drexel's IRB for oversight of this study. You may talk to them at (267) 359-2471 or email HRPP@drexel.edu for any of the following:

- Your questions, concerns, or complaints are not being answered by the research team.

- You cannot reach the research team.
- You want to talk to someone besides the research team.
- You have questions about your rights as a research subject.
- You want to get information or provide input about this research.

6. Why are we doing this research?

Children with developmental delays need treatment. It is important to give children the chance to get help. We are doing this research to learn if health care providers can help parents identify developmental delays early so that they can receive intervention. We want to help more children receive accurate detection of delays so that they may receive early intervention services.

7. How long will the research last?

We expect that you will be in this research study for up to 5 years.

8. How many people will be studied?

We expect to screen up to 10,000 children. We expect up to 500 children to come for Step 2. We expect up to 120 children to be in Step 3.

9. What happens if I say yes, I want to be in this research?

There are several steps to this study. Most families will only do Step 1. You have already completed Step 1.

Step 1. The first part of the study happened when you brought your child to a pediatric health care visit. You may have answered questions about your child's development. If you did, your answers were scored. The score tell us if your child is at risk for developmental delays. Your provider may have had concerns about developmental delays. Most children do not show risk for delays, but no screener or healthcare provider is perfect. This means we cannot know for sure if your child has a developmental delay without a clinical evaluation (step 2).

Step 2a: Clinical evaluation (about 2 hours)

- All eligible children who show risk for developmental delays are invited. Risk may be based on screening. Risk also may be from your provider's concerns.
- This appointment is no cost.
- The evaluation will take place at your local university. If you live near Philadelphia, this is Drexel University. If you live in Connecticut, this is the University of Connecticut. If you live near Sacramento, this is the University of California, Davis. Evaluations also may take place with university staff at a nearby location, such as your provider's office.
- We will video record all sessions. All recordings are kept private. We only share recordings with other members of the research team.
- When you come for an appointment you may see several people from the team.
- The evaluation includes testing with your child. It includes questions for the parents. We look at many different behaviors. These include cognitive, language, play, motor, and social behaviors.

APPROVED
Human Research Protection
Protocol # 1607004653-R004
Approval Date: 07/24/2020
Expiration Date: 07/23/2021

- One or more members of the team will test and observe your child using toys.
- Others will talk with you about your child and ask you to fill out questionnaires.
- We will measure your child's head size using a measuring tape.
- We may all be in the same room. Or we may separate so one parent can talk without your child listening. This is your choice. You will be able to see and interact with your child.
- One member of the evaluation team is a professional. This person may be a licensed clinical psychologist. They also may be a certified school psychologist or a developmental pediatrician. The others are graduate students, staff, and research assistants. These members work under the lead clinician's supervision.
- You and your child can take as many breaks as needed.
- You are encouraged to bring snacks and toys for your child during breaks.

Short break (about 45 minutes)

Step 2b: Feedback (about 30 to 45 minutes)

- In the last part of the appointment, the team will talk with you about the results.
- We will tell you if your child has a clinical diagnosis.
- Some children will not have a diagnosis. If your child does not have a diagnosis, we will still talk with you about your child's strengths and weaknesses.
- We will answer all of your questions.
- You can call us after the appointment if you think of questions you want to ask.
- We will mail you a report explaining all of the testing.
    - This comes to you about 4-6 weeks after your appointment.
    - The report will include a list of local and online resources for you to find help for your child.
    - If you sign a Release of Information form, we can send a copy of the report to your child's provider. We also can send the report to other doctors or therapists.
    - We will only share your child's report if you ask us to. We will only share the report with the specific people you name on the Release of Information form.

The combined length of all parts of step 2 (clinical evaluation, break, and follow-up) is 3-4 hours.

Step 3a: Early intervention (about 20 hours/week for 12 months)

- Some children who receive a clinical diagnosis will be invited to receive early intervention.
- This involves approximately four to five sessions per week for one year. Each session may last about 3-5 hours.
- These sessions should not incur costs the family would not have had without the study.
    - Insurance may be billed for sessions. If families need help paying co-pays, the research study will assist them.
- These sessions will take place at your local university, in your home, or a community setting. You can tell us where you prefer to have intervention.

APPROVED
Human Research Protection
Protocol # 1607004653-R004
Approval Date: 07/24/2020
Expiration Date: 07/23/2021

- We will video record all sessions. All recordings are kept private. We only share recordings with other members of the research team.
- Your child may work with different therapists.
- The intervention sessions include teaching your child skills. Therapists use play to teach children.
- The therapists will look at your child's skills and behavior at the start of therapy. We will talk with you about the goals of therapy for your child. You will tell us what goals are important to your family. We encourage 20 hours per week of therapy. As needed, we will talk about how many hours per week work best for your family.
- You can choose to stay in the same room. Or you can view the session from an observation room.
- The therapists are supervised by an expert in this type of treatment. Therapists may be graduate or undergraduate students. They also may be staff.
- Your child can take as many breaks as needed during sessions.
- You can have snacks and toys for your child during breaks.

Step 3b: Intervention evaluation(s) (about 2 hours)

- All children who receive intervention will be invited for evaluations during and/or after intervention. Based on your child's age, you will have 1-3 evaluations. The last evaluation will take place when your child is about 5 years old.
- See Step 2 for more about evaluations.
- We will send you results after each evaluation. We will send this by mail. After some evaluations, this will be a full clinical report. After other evaluations, this will be a summary. This summary will update diagnosis and recommendations as needed.
    - All results are mailed about 4-6 weeks after your appointment.
    - The report will include a list of local and online resources for you to find help for your child if needed.
    - If you sign a Release of Information form, we can send a copy of the report to your child's provider. We also can send the report to other doctors or therapists.
    - We will only share your child's report if you ask us to. We will only share the report with the specific people you name on the Release of Information form.

10. What are my responsibilities if I take part in this research?

If you take part in this research, it is very important that you tell your study researcher right away if you or your child have a complication or injury.

11. What happens if I do not want to be in this research?

You may decide not to take part in the research and it will not be held against you. You are entitled by law to receive early intervention services if your child meets eligibility requirements, even if you do not take part in this study.

APPROVED
Human Research Protection
Protocol # ***1607004653-R004***
Approval Date: ***07/24/2020***
Expiration Date: ***07/23/2021***

12. What happens if I say yes, but I change my mind later?

Participation in research is voluntary. You have the right to refuse to be in this study. If you decide to be in the study and change your mind, you have the right to stop at any time. Whatever you decide, you will not lose any benefits to which you are otherwise entitled. If you decide not to participate, your relationship with your pediatric provider will not change in any way. You can stop participating at any time without having to explain and with no penalty. If you decide to leave the research, contact the researcher so that the researcher can document that you stopped being in the study. If you stop being in the research, already collected data may not be removed from the study database. But we will not collect new data if you stop the study.

13. Is there any way being in this study could be bad for me?

There are few potential risks of the study. We will only use child-safe toys in this study. The sessions described above pose no foreseeable risks. Some parents may experience discomfort when completing questionnaires. Some feel discomfort when learning that their child has developmental delays. However, the benefits of early identification and intervention far outweigh the risk of distress. If you experience any distress, we will talk to you about it. The researchers also will help you find a counselor. If you decide after the appointment that you want to talk to someone, you can call us. We can answer your questions after the appointment is over. We can help you find a counselor at any time. You will be responsible for all costs of counseling or other treatment. Some children get tired or frustrated. We will give as many breaks as needed.

14. Do I have to pay for anything while I am on this study?

There is no cost to you for participating in this study. If your child is in Step 3, your insurance may be billed for sessions. If you need help paying co-pays, we will help you.

15. Will being in this study help me in any way?

There may be no direct benefit to you from participating in this study. However, there are large benefits of finding delays early and receiving early intervention. Additionally, early diagnosis will help your child get intervention services. Your child may get one year of treatment. Specific recommendations will be provided in writing. We will give you a list of local resources. We also will suggest some websites and books. For those children who do not require intervention, there is benefit to society by allowing us to study typical development. If you come for Step 2, parking or compensation for public transportation to your local university will be provided. We also provide compensation for completing in-person sessions (Step 2). This compensation includes $50 and the written report explaining the evaluation.

16. What happens to the information we collect?

We will keep your records private to the extent allowed by law. Your name, your child's name, and other facts that might identify you will not appear when we share data with other researchers, present this study, or publish its results. The findings will be summarized and reported in group form. You will not be identified personally. We intend to keep all information collected as research records at Drexel University, the University of Connecticut, and the University of California, Davis. The research teams at these universities may see your information. Your child's pediatric provider may keep a copy of this questionnaire in his/her records. Your child's

APPROVED
Human Research Protection
Protocol # ***1607004653-R004***
Approval Date: ***07/24/2020***
Expiration Date: ***07/23/2021***

provider may be notified if you are contacted for parts of the study. We also may share your health information with the Drexel University Institutional Review Board (IRB); National Institute of Mental Health; and the Office for Human Research Protections (OHRP). At the end of the study, we will remove all personal information. Nobody will know who participated. Deidentified data will be stored for use in future research.

This study contributes to a big collection of data across many studies funded by the National Institutes of Health (NIH). The name of this collection is the National Institute of Mental Health Data Archive (NDA). Everything we share will be deidentified. This means all names and other things that identify you and your child will be removed. Nobody will know who is part of the collection. The collection helps researchers learn new and important things about autism and other developmental disorders. We will share information with NDA during and after the study. Experts at NIH will protect the information in this collection very carefully. You may decide now or later that you do not want to share your information using NDA. If so, contact the researchers who conducted this study, and they will tell NDA, which can stop sharing the research information. However, NDA cannot take back information that was shared before you changed your mind. If you would like more information about NDA, this is available online at https://data-archive.nimh.nih.gov/. A description of this clinical trial will be available on http://www.ClinicalTrials.gov, as required by U.S. Law. This Web site will not include information that can identify you. At most, the Web site will include a summary of the results. You can search this Web site at any time.

17. Can I be removed from the research without my OK?

The person in charge of the research study or the sponsor can remove you from the research study without your approval. Possible reasons for removal include if your child has been diagnosed with an autism spectrum disorder before you enroll in the study, or if you or your child are not able to complete other steps of the study due to language barriers or severe disabilities.

18. What else do I need to know?

This study takes place at Drexel University, the University of Connecticut, and the University of California, Davis. Dr. Diana Robins is the head of the study. Dr. Deborah Fein is the lead investigator at the University of Connecticut. Dr. Aubyn Stahmer is the lead investigator at the University of California, Davis. Dr. Dumont-Mathieu is the lead investigator at Connecticut Children's Medical Center. This study is funded by the National Institute of Mental Health.

Parking or compensation for public transportation to Drexel University, the University of Connecticut, or the University of California, Davis will be provided.

The Principal Investigator, Dr. Diana Robins, and co-Investigators Dr. Deborah Fein and Dr. Marianne Barton, have a significant financial interest in the use of the Modified Checklist for Autism in Toddlers (M-CHAT). They co-own, and Dr. Robins is the manager, of M-CHAT, LLC, a for-profit entity. They allow the M-CHAT to be used free of charge for research, teaching, and clinical care. Companies that sell products with the M-CHAT pay royalties.

APPROVED
Human Research Protection
Protocol # 1607004653-R004
Approval Date: 07/24/2020
Expiration Date: 07/23/2021

Federal law provides additional protections of your personal information that are described here.

A. Individually Identifiable Health Information That Will Be Collected

The following personal health information about you will be collected and used during the research study and may be given out to others:

- Your full name, address, telephone numbers, email addresses, date of birth, and place of birth.
- Personal and family medical history.
- Information from the clinical evaluation described in this consent form.
- Information learned during telephone calls, surveys, questionnaires and office visits done as part of this research study.
- Information your child's pediatric provider may provide about your child's development.
- Information about financial and social circumstances, or educational level that you provide.

B. Who Will See and Use Your Health Information within Drexel University?

The researcher and other authorized individuals involved in the research study at Drexel University will see your health information and may give out your health information during the research study. These include:

- The researcher and the research staff.
- The institutional review board and their staff.
- Legal counsel.
- Research office and compliance staff.
- Officers of the organization and other people who need to see the information in order to conduct the research study or make sure it is being done properly.

Your health information may be disclosed or transmitted electronically.

C. Who Else May See and Use your Health Information?

Other persons and organizations outside of Drexel University may see and use your health information during this research study. These include:

- Governmental entities that have the right to see or review your health information, such as The Office for Human Research Protections.
- Research staff at the University of Connecticut will see health information for participants at their site.
- Research staff at the University of California, Davis will see health information for participants at their site.
- Pediatric providers and staff at the community settings where this research study will take place.
- The sponsor of this research study and persons that the sponsor may hire to work on the research study. The sponsor is the National Institute of Mental Health.

APPROVED
Human Research Protection
Protocol # **1607004653-R004**
Approval Date: **07/24/2020**
Expiration Date: **07/23/2021**

If your health information is given to someone not required by law to keep it confidential, then that information may no longer be protected, and may be used or given out without your permission.

D. If you do not want to give authorization to use your health information

You do not have to give your authorization to use or give out your health information. However, if you do not give authorization, you cannot participate in this research study.

E. How to cancel your authorization

At any time you may cancel your authorization to allow your health information to be used or given out by sending a written notice to Human Research Protection at at1505 Race Street, Bellet Building 7th Floor, Philadelphia, Pennsylvania, 19102. If you leave this research study, no new health information about you will be gathered after you leave. However, information gathered before that date may be used or given out if it is needed for the research study or any follow-up.

F. When your authorization ends

Your authorization to use and give out health information will continue until you withdraw or cancel your authorization.

After the research study is finished, your health information will be maintained in a research database. Drexel University shall not re-use or re-disclose the health information in this database for other purposes unless you give written authorization to do so. However, the Drexel University Institutional Review Board may permit other researchers to see and use your health information under adequate privacy safeguards.

G. Your right to inspect your medical and research records

You have the right to look at your medical records at any time during this research study. However, the researcher does not have to release research information to you if it is not part of your medical record.

APPROVED
Human Research Protection
Protocol # ***1607004653-R004***
Approval Date: ***07/24/2020***
Expiration Date: ***07/23/2021***

Signature Block for Children

Your signature documents your permission for the named child to take part in this research.

DO NOT SIGN THIS FORM AFTER THIS DATE → July 23, 2021

____

Printed name of child

____ ____________________

Signature of parent or individual legally authorized to consent to the child's general medical care — Date

- ❑ Parent
- ❑ Individual legally authorized to consent to the child's general medical care (See note below)

____

Printed name of parent or individual legally authorized to consent to the child's general medical care

Note: Investigators are to ensure that individuals who are not parents can demonstrate their legal authority to consent to the child's general medical care. Contact legal counsel if any questions arise.

____ ____________________

Signature of parent — Date

____

Printed name of parent

If signature of second parent not obtained, indicate why: (select one)

- ❑ The IRB determined that the permission of one parent is sufficient.
- ❑ Second parent is deceased
- ❑ Second parent is unknown
- ❑ Second parent is incompetent
- ❑ Second parent is not reasonably available
- ❑ Only one parent has legal responsibility for the care and custody of the child

____ ____________________

Signature of person obtaining consent — Date

____

Printed name of person obtaining consent — Form Date

APPROVED
Human Research Protection
Protocol # **1607004653-R004**
Approval Date: **07/24/2020**
Expiration Date: **07/23/2021**

Signature Block for Capable Adult

Your signature documents your permission to take part in this research.

DO NOT SIGN THIS FORM AFTER THIS DATE → July 23, 2021

______________________________ _______________
Signature of subject — Date

____
Printed name of subject

______________________________ _______________
Signature of person obtaining consent — Date

____
Printed name of person obtaining consent — Form Date

APPROVED
Human Research Protection
Protocol # ***1607004653-R004***
Approval Date: ***07/24/2020***
Expiration Date: ***07/23/2021***
